# Is foliar tissue drying and grinding required for reliable and reproducible extraction of total inorganic nutrients? A comparative study of three tissue preparation methods

**DOI:** 10.3389/fpls.2022.1012764

**Published:** 2022-11-18

**Authors:** Rakesh Minocha, Stephanie Long

**Affiliations:** USDA Forest Service, Northern Research Station, Durham, NH, United States

**Keywords:** conifers, hardwoods, maple, microwave digestion, oak, pine, spruce

## Abstract

In response to abiotic and biotic stress or experimental treatment(s), foliar concentrations of inorganic nutrients and metabolites often change in concert to maintain a homeostatic balance within the cell’s environment thus allowing normal functions to carry on. Therefore, whenever possible, changes in cellular chemistry, metabolism, and gene expressions should be simultaneously evaluated using a common pool of tissue. This will help advance the knowledge needed to fill the gaps in our understanding of how these variables function together to maintain cellular homeostasis. Currently, foliar samples of trees for total inorganic nutrients and metabolic analyses are often collected at different times and are stored and processed in different ways before analyses. The objective of the present study was to evaluate whether a pool of wet (previously frozen) intact tissue that is used for metabolic and molecular work would also be suitable for analyses of foliar total inorganic nutrients. We compared quantities of nutrients extracted from wet-intact, dried-intact, and dried-ground tissues taken from a common pool of previously frozen foliage of black oak (*Quercus velutina* L.), sugar maple (*Acer saccharum* Marshall), red spruce (*Picea rubens* Sarg.), and white pine (*Pinus strobus* L.). With a few exceptions in the case of hardwoods where concentrations of total Ca, Mg, K, and P extracted from wet-intact tissue were significantly higher than dry tissue, data pooled across all collection times suggest that the extracted nutrient concentrations were comparable among the three tissue preparation methods and all for species. Based on the data presented here, it may be concluded that drying and grinding of foliage may not be necessary for nutrient analyses thus making it possible to use the same pool of tissue for total inorganic nutrients and metabolic and/or genomic analyses. To our knowledge, this is the first report on such a comparison.

## 1 Introduction

Traditionally, leaf samples have been prepared by oven-drying followed by grinding the tissue to powder before analyzing for total inorganic nutrients (Pequerul et al., 1993; Linder, 1995; Hansen et al., 2009; Ribeiro et al., 2022; Richard, 2022; Wen et al., 2022). This required the collection and processing of relatively large quantities of green leafy tissue. Another reason for using a larger sample size in many studies was the need to cut the cost of analyses by pooling same-age leaf tissues either from several branches of a single tree or from 4 or 5 trees per species into one composite homogeneous sample (Minocha et al., 2000; Elvir et al., 2010). In several other studies where stand-level estimates were needed, even 4-5 age classes were pooled into a single sample before analyses (Dauer and Perakis, 2014). However, recent investigations in our laboratory (personal communications Alex Young and others) and previously published reports (Lewis et al., 2000; Liu et al., 2012; Niinemets et al., 2015) have revealed that the concentrations of not only total inorganic nutrients but other physiological and metabolic parameters also vary significantly within the depth of a crown. According to these records, even when branches were sampled from within the mid-canopy area for a given study, but the distance from the top of the canopy varied more than two meters for the same trees, the concentrations of nutrients and metabolites in the foliage would be significantly different. Reported literature shows that under abiotic and biotic stress dilute acid-soluble inorganic nutrients and metabolites changed in concert in order to maintain homeostatic balance within cells (Minocha et al., 1997; Bubier et al., 2011; Schaberg et al., 2011; Ma et al., 2020; Mcdermot et al., 2020; Minocha et al., 2021; Blagden et al., 2022; Majumdar et al., 2022). Thus, the current practices of pooling foliar tissue (as described above) followed by drying and grinding before total inorganic nutrients analyses, though economical, are not the best if the goal is to advance our understanding of the collaborative role of nutrients alongside metabolites in maintaining homeostasis within the plant tissue under study. Therefore, ideally, an overall smaller and more homogeneous pool of tissue (collected from a well-defined area of the canopy for all the trees in a study) should be divided for simultaneously analyzing the relationship of metabolites and nutrients to evaluate the role of these variables in growth maintenance and stress regulation.

Presently, tissue samples for metabolic and molecular measurements are often taken as small subsamples (25 mg to 200 mg of fresh weight) from a common pool so the data gathered from these various analyses can be used to understand metabolic regulation within cells under the study conditions. The tissue for such work is often collected in small microfuge tubes and is either flash-frozen in the field or kept on ice until arrival in the laboratory and then frozen at -20°C until analyses. It would be ideal to use a subsample from the same tissue pool for total inorganic nutrient analyses because this will not only allow for studying the relationships among all these variables under the same conditions but also increase our knowledge regarding how they all function together. The goal of the present study was to take a subsample for inorganic nutrient analyses from a pool of tissue that was collected for metabolic and molecular analyses in order to evaluate whether total inorganic nutrients could be extracted equally efficiently using wet (previously frozen) leaf discs when compared with dried and often ground tissue used currently.

We compared the quantities of total inorganic nutrients extracted directly from: 1) wet (not oven-dried and not ground); 2) oven-dried intact; and 3) oven-dried ground tissues using previously frozen stocks of black oak (*Quercus velutina* L.), sugar maple (*Acer saccharum* Marshall), red spruce (*Picea rubens* Sarg.), and white pine (*Pinus strobus* L.) foliage. We hypothesized that the tissue prepared by any one of the three ways would extract identical amounts of inorganic nutrients. This experiment was repeated over four seasons with four trees per species for assessing whether this data would be reproducible and reliable for use at any time of the year.

## 2 Materials and methods

### 2.1 Site description

Foliar samples of red spruce, white pine, black oak, and sugar maple were collected from trees growing at sites owned and managed by the University of New Hampshire, Durham, NH, USA. Black oak and sugar maple samples were collected from East Foss Farm, (Durham, NH, N43° 07’ 08” W70° 56’ 12”), red spruce from the Kingman Farm plantation (Madbury, NH, N43° 10’ 21” W70° 55’ 45”), and white pine from the Woodman Horticultural Farm (Durham, NH, N43° 08’ 52” W70° 56’ 26”). More information for each site is available at https://colsa.unh.edu/woodlands/managed-properties. Climate data for the town of Durham, NH, and Madbury, NH is available at https://www.usclimatedata.com/climate/durham/new-hampshire/united-states

### 2.2 Foliar sampling

Samples were collected four times (June and September of 2014, and February and April of 2015) for conifer trees and three times for hardwoods (June and September of 2014, and July of 2015). Both current-year (CY) and previous-year (PY) foliage were sampled for the red spruce at all four collection times. However, whereas white pine CY was sampled at all four collection times, PY foliage could only be sampled in June of 2014 due to defoliation caused by needle-cast fungi. Samples were collected from the same general area at mid-canopy using a pole pruner, and both CY and PY foliage was collected from the same branchlet. Foliage remained intact on the woody stems and was placed in a cooler for transport back to the laboratory (US Forest Service, Louis C. Wyman Forestry Sciences Lab, Durham, NH) and processed immediately upon arrival. For each collection, four trees per species (n = 4) were sampled except in June 2014 where n = 1 for all species.

Visually healthy leaves from several branchlets were chosen from each sample. For each tree, a pool of approximately five grams (g) of leaf tissue was collected. Hardwood samples were collected as leaf disks using a paper punch (6.4 mm in diameter) avoiding the thick midrib. The foliage of conifer trees was finely chopped into small (1-2 mm) pieces using scissors. Each sample was thoroughly mixed to create a homogeneous pool which was then kept frozen at -20°C until the time of analysis. For the three tissue preparation methods, pooled tissue for each tree was thawed and subsamples were taken and processed as described below. For dried-ground and dried-intact tissue samples, approximately three g of tissue was placed in a clean Qorpak jar (Berlin Packaging, Chicago, IL, USA) and oven-dried at 70°C for seven days. Initial fresh weights and final dry weights were recorded to calculate the percent moisture content. For the dried-ground method, approximately one g of the dry material was ground for one minute using a SPEX SamplePrep shatterbox (Metuchen, NJ, USA) with a small volume ball mill to produce a fine homogeneous powder. The rest of the dry tissue was used for the dried-intact method. All dried samples were stored at room temperature (~20°C). The remaining pool of thawed tissue was returned to the freezer where it remained until thawed again just before being weighed for analysis. Except for one time period of June 2014, four replicate trees were used for each kind of tissue preparation to make sure the data are reproducible. Two analytical replicates were microwave digested for each sample.

### 2.3 Microwave digestion of foliage

Samples for all three methods were digested in Teflon vessels using a MARS™ Xpress microwave (CEM Corporation, Matthews, NC, USA) following EPA compendium SW-846, Test Method 3052 (Microwave assisted acid digestion of siliceous and organically based metrics, 1996). A day before microwave digestions, the dry samples and National Institute of Standards and Technology (NIST) standards (apple, peach, pine) were oven-dried overnight at 60°C in open Qorpak jars and kept in a desiccator cabinet until ready to be weighed. Samples were thoroughly mixed before weighing. Approximately 100 ± 5 mg dried tissue and 150-160 ± 5 mg fresh tissue (to compensate for the difference (~50-60%) in moisture content between the wet and dry samples) were weighed and transferred to a digest vessel. Each microwave digestion run of 20 Teflon vessels contained a blank, a standard, and analytical replicates for the samples that were placed in the second ring of the turntable to test for any location effects on the extractions. Five ml of concentrated nitric acid (HNO_3_) was added to each vessel and swirled gently making sure no sample was stuck on the sides of the vessel. The mixtures were predigested without caps on the vessels for 15 minutes during which the vessels were swirled two more times. Each vessel was capped with a vent plug and a screw cap. The screw cap was then tightened by inserting it into the capping station with the motor rotating continuously (torque was preset). Vessels were capped and placed into a composite sleeve in the appropriate slot on the turntable by pressing down firmly on the cap to seat the vessel/sleeve in the turntable completely. The power (1600 Watts) used in the method was dependent upon the number of vessels in each run: 60% power for 8-16 vessels; 80% for 17-23; and 100% power for 24-40 vessels. During the run, all composite sleeves were kept in the turntable, even if not holding a vessel. The foliar digestion method used in this study was a ramp-to-temperature method where the temperature was ramped to 200°C over 15 minutes and then was held at 200°C for 15 minutes. After the run, the microwave automatically went to a 10-minute cool-down period. The turntable was then carefully removed to exhaust hood and allowed to cool further for another 15-30 minutes before uncapping the vessels. The vent plug was removed, and any sample present in the plug was carefully rinsed into the vessel with deionized water, the solution was then transferred into a 50 ml acid-washed centrifuge tube. The sides of each vessel were then carefully rinsed (two to three times) with deionized water into the same centrifuge tube not to exceed a total volume of 25 ml. The solution was allowed to cool down to room temperature before adjusting the volume if necessary. The caps were secured and the contents were mixed thoroughly.

### 2.4 Quantitation of nutrients and quality control

Nutrient analyses and quantitation were conducted using a simultaneous axial Inductively Coupled Plasma Optical Emission Spectrophotometer (ICP-OES; Vista CCD, Varian, Palo Alto, CA, USA) and Vista Pro software (version 4.0) following EPA compendium SW-846, Test Method 6010D. National Institute of Standards and Technology (NIST, Gaithersburg, MD, USA) standards for Eastern white pine (SRM 1575A), peach (SRM 1547), and apple (SRM 1515) leaves were all within 5 to 10% of the expected range for each nutrient; Ca and P were higher and Al and Mn were lower than the expected values. There are no registered NIST standards for fresh foliar tissues. However, we also used a groundwood reference for quality control and assurance. This reference material was prepared in our laboratory and has been used routinely for 20 years. For all samples, a standard curve was repeated after every 20 samples, and check standards were run after every recalibration and after every 10 samples.

### 2.5 Statistical analyses

A modified Thompson Tau Test was conducted to remove outliers from the data before conducting statistical tests (Thompson, 1985). For each species, the effect of tissue preparation methods on foliar nutrient concentrations was tested individually using a one-way ANOVA. Means from significant ANOVA were compared using Tukey’s Least Significant Difference (LSD) test (*P* ≤ 0.05). All analyses were done using SYSTAT Version 10.2 for Windows (SYSTAT, Richmond, CA, USA) and Microsoft Excel (Version 2010).

## 3 Results

All nutrient data in this study (whether from fresh or dry tissue) have been presented on a fresh-weight basis because metabolic and molecular data are often presented on a fresh-weight basis. The authors acknowledge that presenting results on either a fresh or dry weight basis would not change the observed trends. The first figure presents an overall summary of method comparisons for each species based on data pooled from all collections made over different seasons. The data on the effects of seasons on quantities of total nutrients extracted by the three methods are presented by following these concentrations in a single representative tree per species (**Figures 2**-**6**). The data on the four replicate trees per species for each season (except for June 2014) show the replicability and reproducibility of the methods (**Supplementary Figures 1**–**10**). Raw data for all micro- and macro-nutrients extracted for collections for the present study are provided in in Supplemental tables 1-6. In general, it was observed that potassium concentrations were most variable among individual trees and with changes in the season.

### 3.1 Hardwoods

**Figure 1:**
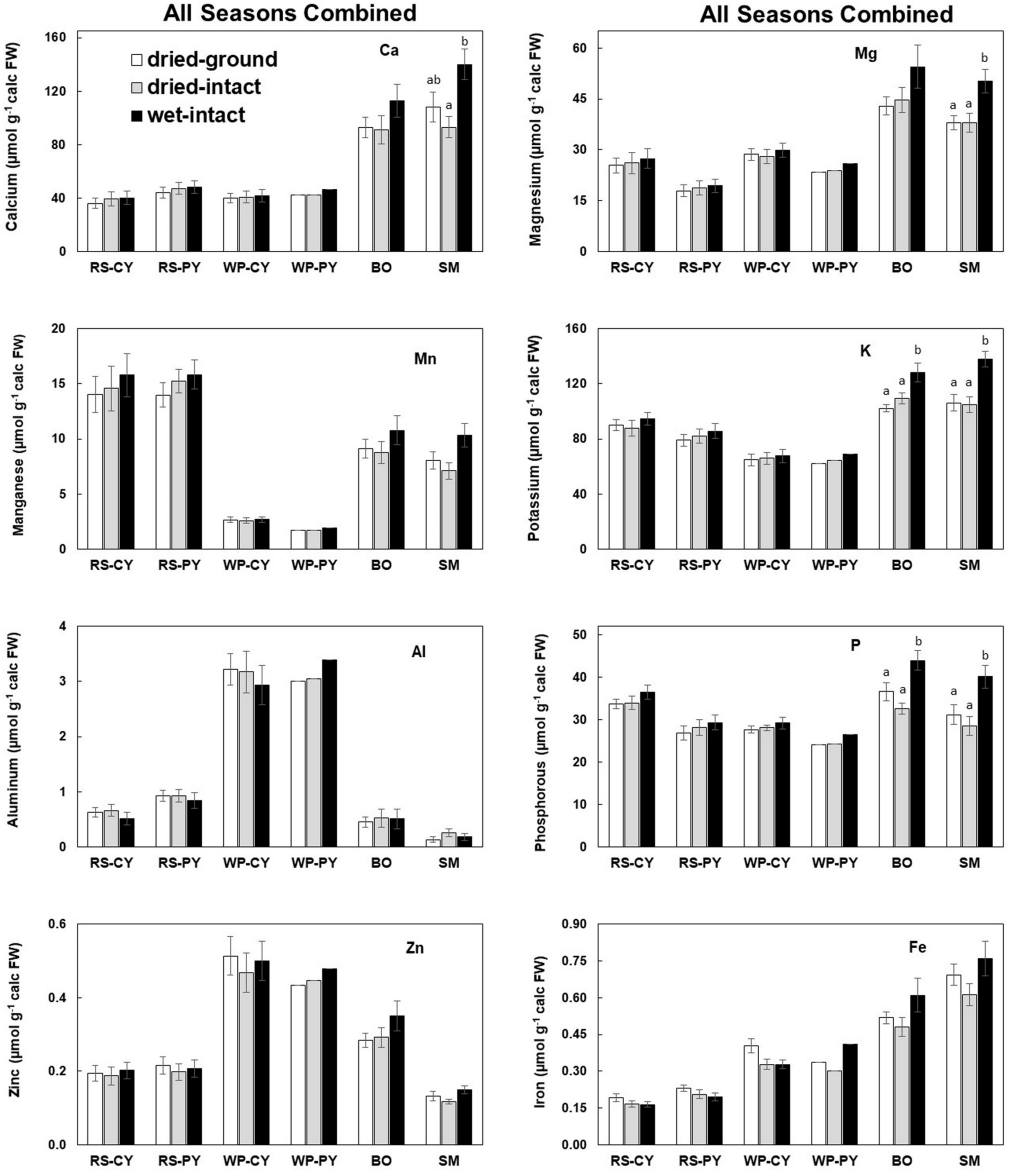
Comparison of three methods of tissue preparation for extraction of total inorganic nutrients from red spruce, white pine, black oak, and sugar maple foliage. Data in each bar are mean ± SE of all samples collected from all seasons during 2014-2015 (six times for conifers and four times for hardwood trees). Replicate numbers for each species across all dates are presented in the order: dried-ground/dried-intact/wet-intact: CY-RS, PY-RS, and CY-WP = 13/10/13; PY-WP = 1/1/1; BO = 9/6/9 and SM = 11/7/11. Letters on top of the bars indicate significant differences among the three methods for each species. P ≤ 0.05. CY stands for Current-Year foliage and PY for Previous-Year.

**Figure 2:**
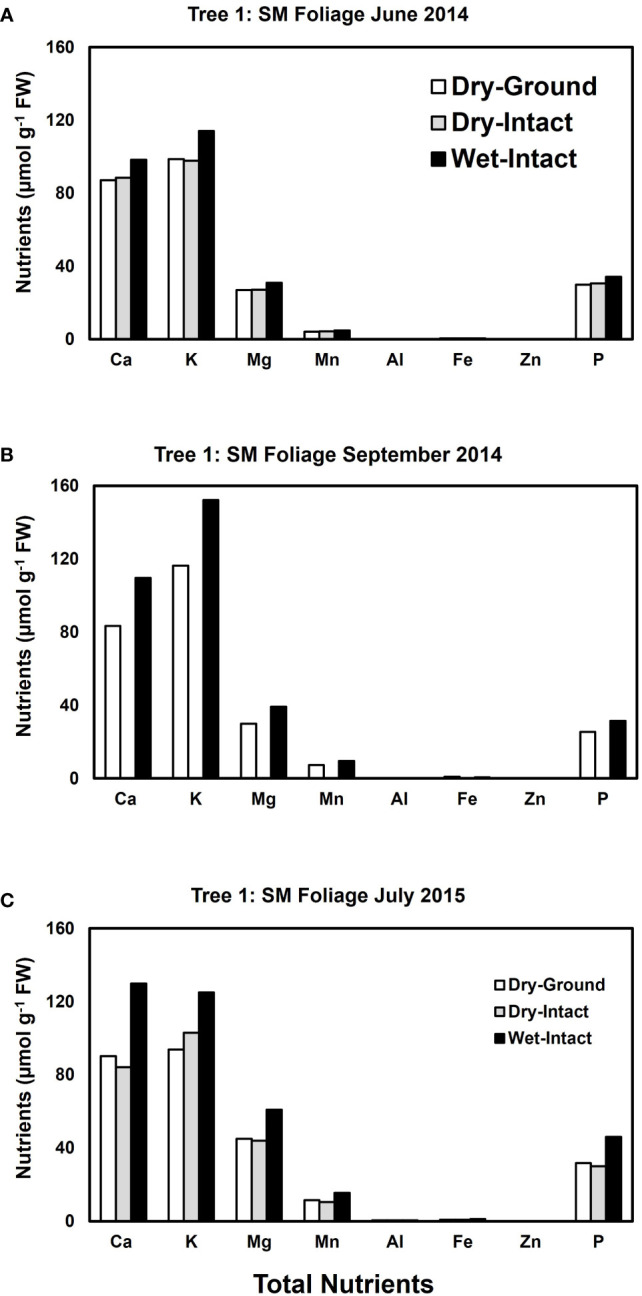
Comparison of three tissue preparation methods for the extraction of total inorganic nutrients from sugar maple foliage. Data presented here are from three collections from a single tree in: June 2014 **(A)**; September 2014 **(B)**; and July 2015 **(C)**.

**Figure 3:**
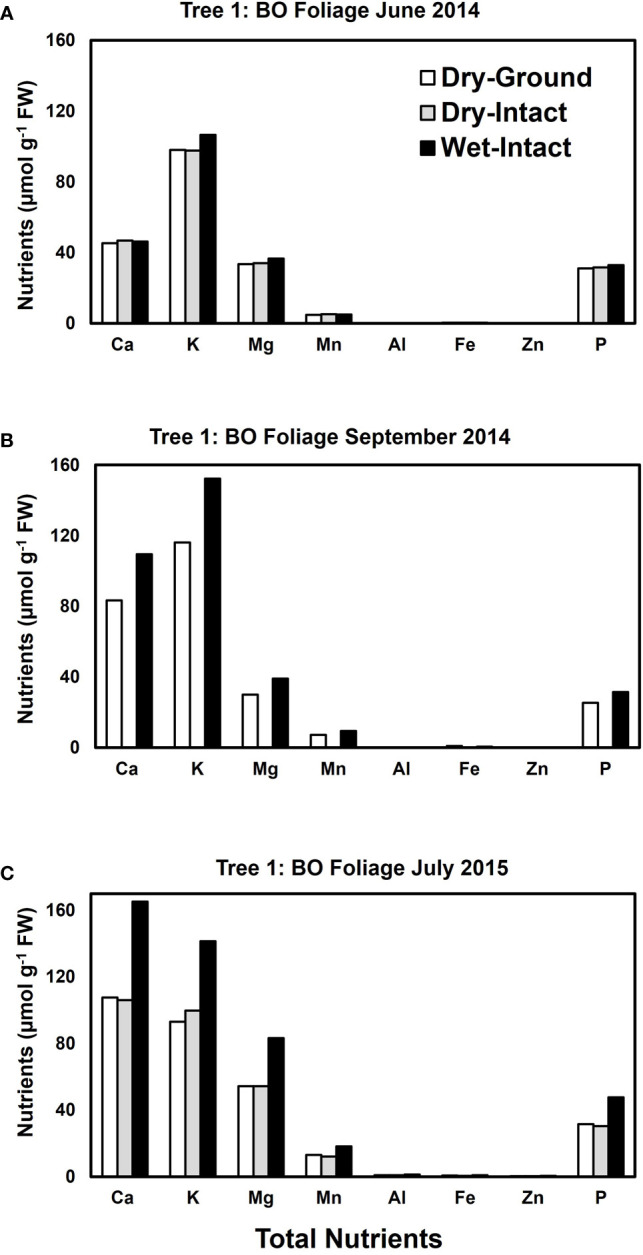
Comparison of three tissue preparation methods for the extraction of total inorganic nutrients from black oak foliage. Data presented here are from three collections from a single tree in: June 2014 **(A)**; September 2014 **(B)**; and July 2015 **(C)**.

Based on pooled data, higher amounts of Ca, Mg, K, and P (*P* ≤ 0.05) were consistently exracted from wet tissue of sugar maple and black oak compared with dry tissue methods (**Figure 1**). The extracted amounts from the dried-ground and dried-intact tissues were the same for both species (**Figure 1**). When a single tree of either maple or oak was tracked over seasons, the concentrations extracted from wet tissue were higher. This trend was more prominent relative to the two dry tissue methods in September 2014 and July 2015 relative to June 2014 (**Figures 2**, **3**). This observed trend with single trees through different seasons repeated itself for the foliage of all four replicate trees of maple and oak that were collected in July 2015 and also in September 2014 (**Supplementary Figures 1–4**) demonstrating the reliability and reproducibility of both wet and dry-intact tissue extraction methods for quantitation of total nutrients in sugar maple and balck oak foliage.

### 3.2 Conifers

**Figure 4:**
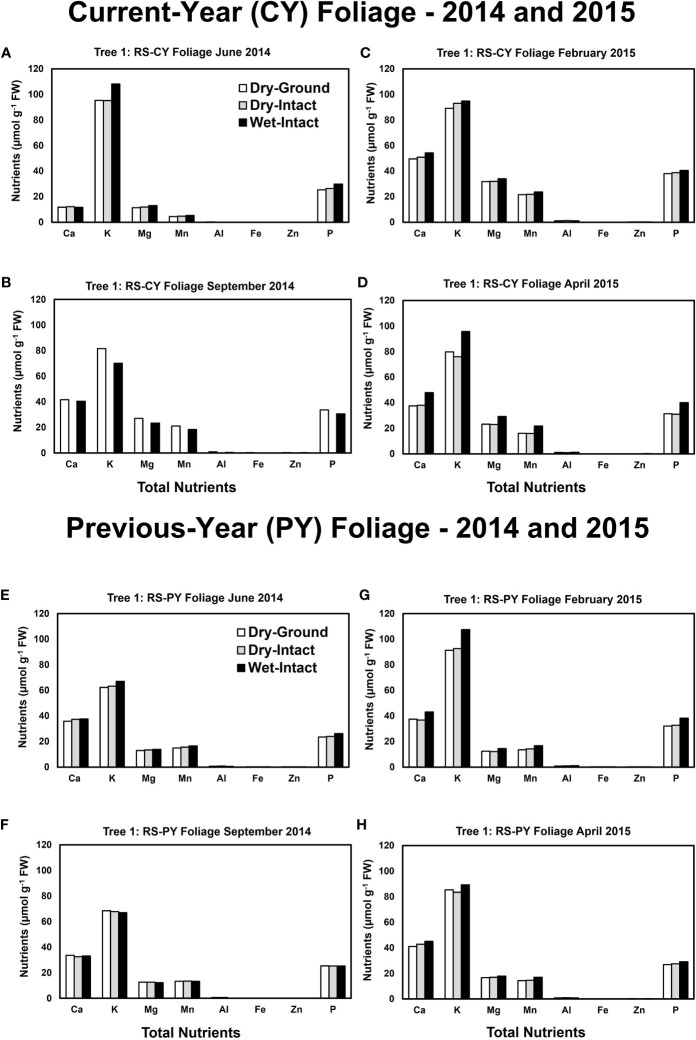
Comparison of three tissue preparation methods for extraction of total inorganic nutrients from red spruce CY foliage **(A-D)** and PY foliage **(E–H)** both collected from the same branchlet. Data presented here are from four collections from a single tree in: June 2014 **(A, E)**; September 2014 **(B, F)**; February 2015 **(C, G)**; and April 2015 **(D, H)**.

Based on pooled data, there were no significant differences between the concentrations of nutrients extracted by the three methods in CY or PY red spruce foliage (**Figure 1**). When a single tree was followed over different seasons, it was observed that with one exception of September 2014, wet-intact CY and PY foliage of red spruce showed a trend towards slightly higher concentrations of total inorganic nutrients than those extracted from dried tissue at all collection times (**Figure 4**). For both needle age classes, the extracted amounts from dried-ground and dried-intact tissues were comparable (**Figure 4**). Similar trends were observed for each of the four replicate trees sampled in September 2014 (**Supplementary Figure 5**), February 2015 (**Supplementary Figure 6**), and April 2015 (**Supplementary Figure 7**) again validating the reproducibility and reliability of the results.

**Figure 5:**
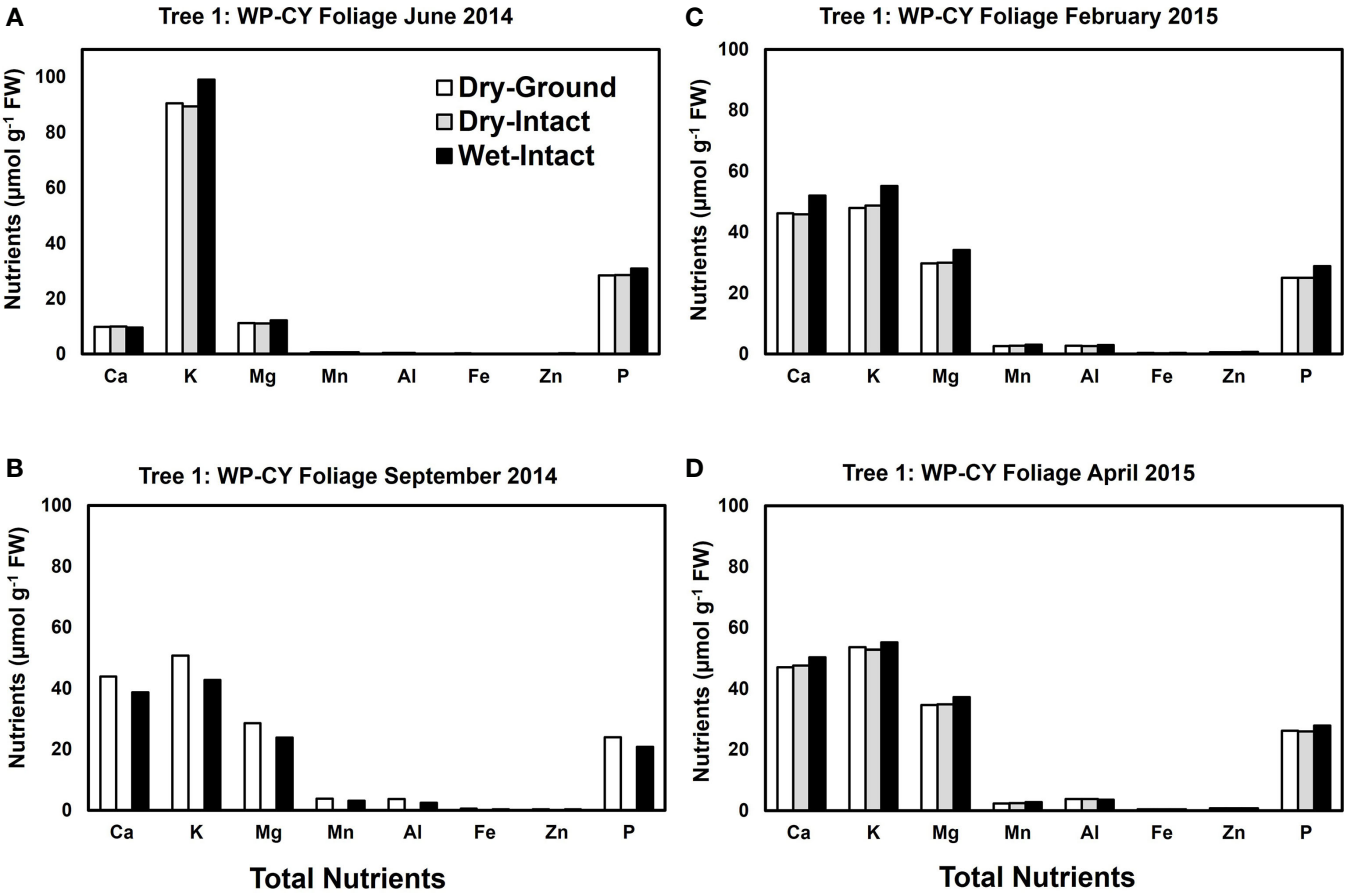
Comparison of three tissue preparation methods for extraction of total inorganic nutrients from CY foliage of white pine. Data are from four collections from a single tree in: June 2014 **(A);** September 2014 **(B)**; February 2015 **(C)**; and April 2015 **(D)**.

**Figure 6:**
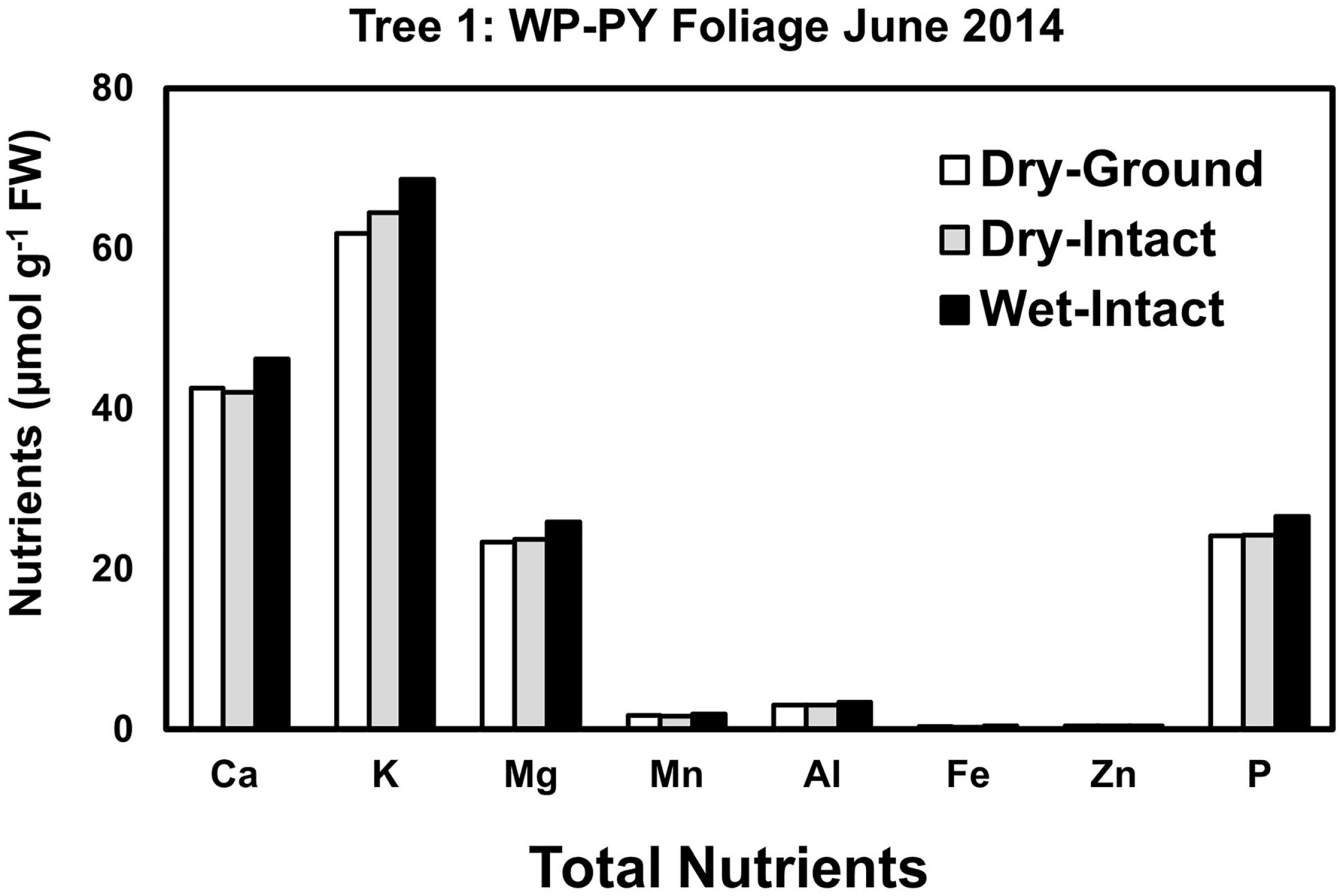
Comparison of three different tissue preparation methods for extraction of total inorganic nutrients from PY foliage of white pine. Samples were collected from a single tree in June 2014. White pine PY foliage was not available after June 2014 due to defoliation caused by a region-wide infestation of needle cast fungi.

Based on pooled data, Similar to red spruce, white pine CY and PY (June 2014 collection only) foliage also revealed no significant differences in the concentrations of nutrients extracted by any of the three methods. (**Figure 1**). When a single tree was followed over different seasons, it was observed that with one exception in September 2014, concentrations of total inorganic nutrients extracted from wet-intact CY foliage of white pine were slightly higher than those extracted from dried tissues at all collection times (**Figure 5**). Results similar to those observed for CY foliage in June 2014 were also observed for PY foliage collected only one time in June 2014. (**Figure 6**). In all cases, extracted amounts from dried-ground and dried-intact tissues were comparable (**Figures 5**, **6**). Similar trends observed in four replicate trees of CY foliage sampled in September 2014 (**Supplementary Figure 8**), February 2015 (**Supplementary Figure 9**), and April 2015 (**Supplementary Figure 10**) demonstrated the consistency and reproducibility of the results.

## 4 Discussion

Previous research from our laboratory has shown that cellular concentrations of dilute acid (5% perchloric acid soluble) extractable inorganic nutrients and metabolites from the same pool of wet (previously frozen or fresh) foliage of plants/trees often changed in concert and many showed strong relationships with each other in response to abiotic and biotic stress or with experimental treatments. Some examples of such studies are 1) soil-based N supplementation of the mixed-hardwood forest at Harvard Forest, MA affected both nutrient and metabolic profiles of red pine, black and red oak, and red maple trees (Minocha et al., 2000; Bauer et al., 2004; Minocha et al., 2015b); 2) Al toxicity originating from acidic deposition affected soil Al/Ca ratios and homeostatic balance in red spruce and sugar maple tissues in Northeast New England (Minocha et al., 1997; Wargo et al., 2002); 3) elevation-dependent changes occurring simultaneously in cellular soluble nutrients and metabolites were demonstrated in tree species growing in different countries (Minocha et al., 2010; Minocha et al., 2021). Elevation levels are known to be related to air pollution levels (higher elevations are more exposed to acidic deposition); 4) Climate change also influences foliar nutrition and metabolism simultaneously as was shown in red maple (*Acer rubrum*) trees exposed to soil warming with or without induced freeze-thaw cycles in a northern hardwood forest (Blagden et al., 2022); and 5) *Rhizoctonia solani*’s damage to sugar beet increased in the presence of *Leuconostoc mesenteroides* as revealed by altered roots nutrient and metabolite levels in the infected roots (Majumdar et al., 2022). These authors suggested a common role of inorganic nutrients and primary metabolism in all host-plant fungal and bacterial pathogens besides the well-known role of unique pathways and genes in the pathogens towards maintaining successful pathogenesis and the development of disease symptoms. These observations regarding the interactive changes between nutrients and metabolites listed above were made possible mainly because our studies used the same pool of tissue for all analyses. To date our group has only tested these relationships with dilute-acid soluble fractions of nutrients and metabolites that we believe are more readily bioavailable portions under stressful conditions. This portion of nutrients contains Ca present as CaOx with 2 N HCl or 5% PCA (Minocha et al., 2015a). However, scientists in the biogeochemistry field have always made use of the total inorganic nutrient concentrations and analyze these by first drying and grinding the leaf tissue (Crim et al., 2019; Mcdermot et al., 2020; Arseneau et al., 2021; Hong et al., 2022). Therefore, to bridge this gap and to be able to make more direct comparisons among both types of reported studies, the present study was designed to explore an efficient and reproducible way to conduct total inorganic nutrient analyses using a subsample of the same pool of wet tissue as is often used for metabolomic and genomic analyses. Our findings show that similar, if not greater, amounts of total inorganic nutrients can be extracted from wet compared to dried tissue samples. Further, the efficiency of this method was comparable regardless of when foliar samples were collected. Consistency in trends seen in four replicate trees of each species validated the reproducibility of the results of this study. The data presented here offer another tool for advancing our understanding of how these variables function together to maintain cellular homeostasis. To our knowledge, the current study is the first to compare different tissue preparation methods for the optimum extraction of total nutrients.

## 5 Conclusions

In summary, regardless of tree species, season, needle age, or variations among replicate trees, the concentrations of total inorganic nutrients extracted from dried-ground and dried-intact foliage were comparable. Amounts of Ca, Mg, K, and P extracted from wet-intact tissue were generally higher than either dried-intact or dried-ground tissue for all species, however the differences were only significant for the hardwoods. These consistent differences in extracted amounts between wet and dried tissue could be the outcome of different ways of handling and processing dried vs. wet samples. Thus, if one routinely uses either wet or dried tissue, the results should be reproducible and consistent over time. Used as a tool, data gathered from the same small pool of tissue for nutrients, metabolites, and genomics could speed up the advancement our understanding of the mechanisms regulating cellular health and may also help with accurate estimations of canopy performance when needed.

## Data availability statement

The original contributions presented in the study are included in the article/**Supplementary Material**. Further inquiries can be directed to the corresponding author.

## Author contributions

RM and SL contributions: both authors made equal contribution to this work. While RM led the writing part, SL led the analytical and data processing part. All authors contributed to the article and approved the submitted version.

## Funding

Funding for this work was provided solely by the USDA Forest Service, Northern Research Station.

## Acknowledgments

The authors are grateful to Gloria Quigley and Kenneth Dudzik for their help with the collection and analysis of samples. The authors would also like to thank Emeritus Prof. Subhash Minocha for reviewing the manuscript.

## Conflict of interest

The authors declare that the research was conducted in the absence of any commercial or financial relationships that could be construed as a potential conflict of interest.

## Publisher’s note

All claims expressed in this article are solely those of the authors and do not necessarily represent those of their affiliated organizations, or those of the publisher, the editors and the reviewers. Any product that may be evaluated in this article, or claim that may be made by its manufacturer, is not guaranteed or endorsed by the publisher.

## References

[B1] Arseneau J. Bélanger N. Ouimet R. Royer-Tardif S. Bilodeau-Gauthier S. Gendreau-Berthiaume B. . (2021). Wood ash application in sugar maple stands rapidly improves nutritional status and growth at various developmental stages. For. Ecol. Manage. 489, 119062. doi: 10.1016/j.foreco.2021.119062

[B2] Bauer G. A. Bazzaz F. A. Minocha R. Long S. Magill A. Aber J. . (2004). Effects of chronic n additions on tissue chemistry, photosynthetic capacity, and carbon sequestration potential of a red pine (*Pinus resinosa* ait.) stand in the NE united states. For. Ecol. Manage. 196, 173–186. doi: 10.1016/j.foreco.2004.03.032

[B3] Blagden M. Harrison J. L. Minocha R. Sanders-Demott R. Long S. Templer P. H. (2022). Climate change influences foliar nutrition and metabolism of red maple (*Acer rubrum*) trees in a northern hardwood forest. Ecoshere 13, e03859. doi: 10.1002/ecs2.3859

[B4] Bubier J. L. Smith R. Juutinen S. Moore T. R. Minocha R. Long S. . (2011). Effects of nutrient addition on leaf chemistry, morphology, and photosynthetic capacity of three bog shrubs. Oecologia 167, 355–368. doi: 10.1007/s00442-011-1998-9 21544572

[B5] Crim P. M. Mcdonald L. M. Cumming J. R. (2019). Soil and tree nutrient status of high elevation mixed red spruce (Picea rubens sarg.) and broadleaf deciduous forests. Soil Syst. 3, 80. doi: 10.3390/soilsystems3040080

[B6] Dauer J. M. Perakis S. S. (2014). Calcium oxalate contribution to calcium cycling in forests of contrasting nutrient status. For. Ecol. Manage. 334, 64–73. doi: 10.1016/j.foreco.2014.08.029

[B7] Elvir J. Wiersma G. Bethers S. Kenlan P. (2010). Effects of chronic ammonium sulfate treatment on the forest at the bear brook watershed in Maine. Environ. Monit. Assess. 171, 129–147. doi: 10.1007/s10661-010-1523-3 20556652

[B8] Hansen T. H. Laursen K. H. Persson D. P. Pedas P. Husted S. Schjoerring J. K. (2009). Micro-scaled high-throughput digestion of plant tissue samples for multi-elemental analysis. Plant Methods 5, 12. doi: 10.1186/1746-4811-5-12 19781097 PMC2761891

[B9] Hong D. S. Gonzales K. E. Fahey T. J. Yanai R. D. (2022). Foliar nutrient concentrations of six northern hardwood species responded to nitrogen and phosphorus fertilization but did not predict tree growth. PeerJ 10, e13193. doi: 10.7717/peerj.13193 35474687 PMC9035280

[B10] Lewis J. D. Mckane R. B. Tingey D. T. Beedlow P. A. (2000). Vertical gradients in photosynthetic light response within an old-growth Douglas-fir and western hemlock canopy. Tree Physiol. 20, 447–456. doi: 10.1093/treephys/20.7.447 12651440

[B11] Linder S. (1995). Foliar analysis for detecting and correcting nutrient imbalances in Norway spruce. Ecol. Bull. 44, 178–190. Available at: https://www.jstor.org/stable/20113161

[B12] Liu J. Zhang D. Zhou G. Duan H. (2012). Changes in leaf nutrient traits and photosynthesis of four tree species: effects of elevated [CO2], n fertilization and canopy positions. J. Plant Ecol. 5, 376–390. doi: 10.1093/jpe/rts006

[B13] Majumdar R. Strausbaugh C. A. Galewski P. J. Minocha R. Rogers C. W. (2022). Cell-Wall-Degrading enzymes-related genes originating from rhizoctonia solani increase sugar beet root damage in the presence of leuconostoc mesenteroides. Int. J. Mol. Sci. 23, 1366. doi: 10.3390/ijms23031366 35163289 PMC8835807

[B14] Ma C. Liu H. Chen G. Zhao Q. Guo H. Minocha R. . (2020). Dual roles of glutathione in silver nanoparticle detoxification and enhancement of nitrogen assimilation in soybean (*Glycine max* (L.) Merrill). Environ. Sci. Nano. 7, 1954–1966. doi: 10.1039/D0EN00147C

[B15] Mcdermot C. R. Minocha R. D’amico V. Long S. Trammell T. (2020). Red maple (*Acer rubrum* l.) trees demonstrate acclimation to urban conditions in deciduous forests embedded in cities. PloS One 15, e0236313. doi: 10.1371/journal.pone.0236313 32706781 PMC7380610

[B16] Minocha R. Chamberlain B. Long S. Turplapati S. A. Quigley G. (2015a). Extraction and estimation of the quantity of calcium oxalate crystals in the foliage of conifer and hardwood trees. Tree Physiol. 35, 574–580. doi: 10.1093/treephys/tpv031 25934989

[B17] Minocha R. Contosta A. Lawrence G. B. Kohli R. K. Minocha S. C. Long S. (2021). Effects of regional air pollution and local grazing management on the health of Himalayan cedar (*Cedrus deodara*) and Himalayan spruce (*Picea smithiana*) growing at kufri mountain, HP, India. Forests 12, 400. doi: 10.3390/f12040400

[B18] Minocha R. Long S. Magill A. H. Aber J. Mcdowell W. H. (2000). Foliar free polyamine and inorganic ion content in relation to soil and soil solution chemistry in two fertilized forest stands at the Harvard forest, Massachusetts. Plant Soil 222, 119–137. doi: 10.1023/A:1004775829678

[B19] Minocha R. Long S. Thangavel P. Minocha S. C. Eagar C. Driscoll C. T. (2010). Elevation dependent sensitivity of northern hardwoods to Ca addition at Hubbard brook experimental forest, NH USA. For. Ecol. Manage. 260, 2115–2125. doi: 10.1016/j.foreco.2010.09.002

[B20] Minocha R. Shortle W. C. Lawrence G. B. David M. B. Minocha S. C. (1997). Relationships among foliar chemistry, foliar polyamines, and soil chemistry in red spruce trees growing across the northeastern united states. Plant Soil 191, 109–122. doi: 10.1023/A:1004293523185

[B21] Minocha R. Turlapati S. A. Long S. Mcdowell W. H. Minocha S. C. (2015b). Long-term trends of changes in pine and oak foliar nitrogen metabolism in response to chronic nitrogen amendments at the Harvard forest, MA. Tree Physiol. 35, 894–909. doi: 10.1093/treephys/tpv044 26116927

[B22] Niinemets Ü. Keenan T. F. Hallik L. (2015). A worldwide analysis of within-canopy variations in leaf structural, chemical and physiological traits across plant functional types. New Phytol. 205, 973–993. doi: 10.1111/nph.13096 25318596 PMC5818144

[B23] Pequerul A. Pérez C. Madero P. Val J. Monge E. (1993). “A rapid wet digestion method for plant analysis,” in Optimization of plant nutrition: Refereed papers from the eighth international colloquium for the optimization of plant nutrition, 31 august – 8 September 1992, Lisbon, Portugal. Eds. Fragoso M. A. C. Beusichem M. L. V. Houwers A. (Dordrecht: Springer Netherlands), 3–6.

[B24] Ribeiro S. A. O. Silva Da C. S. Nogueira De Araújo A. R. Garcia E. E. (2022). Solubility of cd, cr, Cu, Ni, and Pb and its correlation with total polyphenols and soluble melanoidins in hot infusions of green and roasted mate. Biol. Trace Elem. Res. doi: 10.1007/s12011-022-03314-3 35689152

[B25] Richard M. (2022). The potential of Kenyan wild plants as supplements for calcium, magnesium, potassium, and aluminum. Int. J. Petro. Chem. Natur. Gas. 2, 69–79.

[B26] Schaberg P. G. Minocha R. Long S. Halman J. M. Hawley G. J. Eagar C. (2011). Calcium fertilization at the Hubbard brook experimental forest increases the capacity for stress tolerance and carbon capture in red spruce (*Picea rubens*) trees during the cold season. Trees 25, 1053–1061. doi: 10.1007/s00468-011-0580-8

[B27] Thompson R. (1985). A note on restricted maximum likelihood estimation with an alternative outlier model. R. Stat. Soc Ser. B Methodol. 47, 53–55. doi: 10.1111/j.2517-6161.1985.tb01329.x

[B28] Wargo P. M. Minocha R. Wong B. L. Long R. P. Horsley S. B. Hall T. J. (2002). Measuring changes in stress and vitality indicators in limed sugar maple on the Allegheny plateau in north-central Pennsylvania. Can. J. For. Res. 32, 629–641. doi: 10.1139/x02-008

[B29] Wen J. Brahney J. Lin Y. Ma Z. Sun N. Zheng J. . (2022). The scaling of leaf nitrogen and phosphorus along a phosphorus availability gradient in a subtropical forest. Plant Ecol. 223, 995–1006. doi: 10.1007/s11258-022-01252-7

