# Supplementary material for: Is foliar tissue drying and grinding required for reliable and reproducible extraction of total inorganic nutrients? A comparative study of three tissue preparation methods
Source: Front Plant Sci. 2022 Nov 18;13:1012764. doi: 10.3389/fpls.2022.1012764 (PMC9716281; doi:10.3389/fpls.2022.1012764)
Supplement: Supplementary file 1 [file DataSheet_1.pdf]

Supplementary Tables 1-6 with Raw Data

Supplemental Table 1. Raw data collected for sugar maple total inorganic nutrients during all the collections are present here. With a few exceptions, these data are the mean of two analytical replicates. "na" denotes data not available.

Sugar Maple Digests				
**Date**	**Element (µmol g^{-1} calc FW)**	**Dried-Ground**	**Dried-leaf discs**	**Wet-leaf discs**
**6/24/2014**				
**Tree 1**	**Ca**	**87.14**	**88.53**	**98.44**
	**K**	**98.77**	**97.85**	**114.04**
	**Mg**	**26.95**	**27.17**	**30.98**
	**Mn**	**4.11**	**4.33**	**4.83**
	**Al**	**0.00**	**0.00**	**0.00**
	**Fe**	**0.56**	**0.46**	**0.53**
	**Zn**	**0.11**	**0.12**	**0.13**
	**P**	**29.96**	**30.66**	**34.20**
**9/25/2014**				
**Tree 1**	**Ca**	**83.44**	**na**	**109.54**
	**K**	**116.22**	**na**	**152.24**
	**Mg**	**29.94**	**na**	**39.12**
	**Mn**	**7.28**	**na**	**9.48**
	**Al**	**0.02**	**na**	**0.00**
	**Fe**	**0.91**	**na**	**0.60**
	**Zn**	**0.09**	**na**	**0.11**
	**P**	**25.33**	**na**	**31.45**
				**n=1**
**Tree 2**	**Ca**	**134.27**	**na**	**183.06**
	**K**	**124.62**	**na**	**167.83**
	**Mg**	**36.13**	**na**	**50.08**
	**Mn**	**6.30**	**na**	**8.41**
	**Al**	**0.00**	**na**	**0.00**
	**Fe**	**0.76**	**na**	**0.55**
	**Zn**	**0.09**	**na**	**0.12**
	**P**	**31.16**	**na**	**41.57**
**Tree 3**	**Ca**	**111.94**	**na**	**138.28**
	**K**	**108.46**	**na**	**126.25**
	**Mg**	**47.52**	**na**	**57.91**
	**Mn**	**13.85**	**na**	**17.06**
	**Al**	**0.01**	**na**	**0.00**
	**Fe**	**0.88**	**na**	**0.62**
	**Zn**	**0.22**	**na**	**0.13**
	**P**	**34.83**	**na**	**41.08**
**Tree 4**	**Ca**	**77.19**	**80.86**	**109.16**
	**K**	**80.87**	**79.08**	**111.01**
	**Mg**	**30.54**	**30.09**	**43.33**
	**Mn**	**6.88**	**6.53**	**9.40**
	**Al**	**0.00**	**0.00**	**0.00**
	**Fe**	**0.58**	**0.54**	**0.77**

	Zn	0.11	0.11	0.14
	P	30.38	29.10	40.78
Tree 5	Ca	198.17	na	179.63
	K	153.15	na	134.53
	Mg	39.76	na	35.86
	Mn	8.58	na	7.63
	Al	0.00	na	0.00
	Fe	0.49	na	0.41
	Zn	0.16	na	0.12
	P	48.34	na	40.24
7/22/2015				
Tree 1	Ca	90.12	84.16	129.87
	K	93.83	103.04	125.04
	Mg	45.01	44.02	60.94
	Mn	11.53	10.54	15.58
	Al	0.43	0.45	0.56
	Fe	0.85	0.82	1.16
	Zn	0.14	0.11	0.18
	P	31.72	30.08	46.09
Tree 2	Ca	113.39	111.72	160.15
	K	103.76	120.58	150.49
	Mg	35.19	36.10	51.17
	Mn	8.05	7.68	10.83
	Al	0.28	0.38	0.39
	Fe	0.70	0.67	0.98
	Zn	0.18	0.15	0.21
	P	23.29	23.24	33.70
Tree 3	Ca	78.86	78.54	115.43
	K	106.86	122.47	157.07
	Mg	47.24	47.83	69.28
	Mn	8.96	8.35	12.12
	Al	0.30	0.37	0.41
	Fe	0.61	0.55	0.83
	Zn	0.10	0.09	0.14
	P	23.21	22.56	33.85
Tree 4	Ca	75.25	74.55	107.04
	K	82.28	96.24	122.58
	Mg	40.13	41.82	57.80
	Mn	6.11	5.79	8.25
	Al	0.24	0.35	0.35
	Fe	0.68	0.65	0.95
	Zn	0.13	0.12	0.17
	P	24.23	24.08	35.13
Tree 5	Ca	140.05	132.96	210.66
	K	99.98	114.42	156.22
	Mg	38.35	38.60	55.89
	Mn	6.86	6.42	10.07
	Al	0.19	0.26	0.30
	Fe	0.61	0.60	0.95
	Zn	0.13	0.12	0.20
	P	40.65	39.39	63.29

Supplemental Table 2. Raw data collected for black oak total inorganic nutrients during all the collections are present here. With a few exceptions, these data are the mean of two analytical replicates. “na” denotes data not available.

Black Oak				
**Collection Date**	**Elements (µmol g-1 calc FW)**	**Dried-Ground**	**Dried-leaf discs**	**Wet-leaf discs**
**6/24/2015**				
**Tree 1**	**Ca**	**45.28**	**46.77**	**46.33**
	**K**	**97.97**	**97.62**	**106.46**
	**Mg**	**33.41**	**33.98**	**36.71**
	**Mn**	**4.82**	**5.09**	**5.00**
	**Al**	**0.00**	**0.00**	**0.00**
	**Fe**	**0.43**	**0.32**	**0.35**
	**Zn**	**0.18**	**0.18**	**0.18**
	**P**	**31.01**	**31.61**	**32.90**
**9/25/2014**				
**Tree 1**	**Ca**	**104.06**	**na**	**119.40**
	**K**	**95.39**	**na**	**130.62**
	**Mg**	**38.62**	**na**	**50.80**
	**Mn**	**7.87**	**na**	**9.25**
	**Al**	**0.50**	**na**	**0.16**
	**Fe**	**0.48**	**na**	**0.52**
	**Zn**	**0.30**	**na**	**0.34**
	**P**	**47.03**	**na**	**54.72**
**Tree 2**	**Ca**	**76.14**	**na**	**74.49**
	**K**	**102.60**	**na**	**109.29**
	**Mg**	**32.49**	**na**	**33.36**
	**Mn**	**7.34**	**na**	**7.09**
	**Al**	**0.46**	**na**	**0.25**
	**Fe**	**0.55**	**na**	**0.54**
	**Zn**	**0.27**	**na**	**0.27**
	**P**	**41.50**	**na**	**41.59**
	**Ca**	**115.44**	**117.13**	**116.34**
**Tree 3**	**K**	**108.04**	**108.14**	**103.85**
	**Mg**	**36.32**	**33.86**	**35.29**
	**Mn**	**9.96**	**9.41**	**10.17**
	**Al**	**0.08**	**0.03**	**0.00**
	**Fe**	**0.44**	**0.43**	**0.42**
	**Zn**	**0.30**	**0.29**	**0.29**
	**P**	**37.93**	**38.40**	**38.93**
	**Ca**	**106.42**	**na**	**96.19**
**Tree 4**	**K**	**114.33**	**na**	**115.98**
	**Mg**	**44.74**	**na**	**42.10**
	**Mn**	**11.34**	**na**	**10.53**
	**Al**	**0.23**	**na**	**0.00**
	**Fe**	**0.49**	**na**	**0.45**
	**Zn**	**0.24**	**na**	**0.22**
	**P**	**43.42**	**na**	**40.85**
**7/22/2015**				

**Tree 1**	**Ca**	**107.68**	**105.99**	**165.17**
	**K**	**93.08**	**99.79**	**141.41**
	**Mg**	**54.30**	**54.27**	**83.24**
	**Mn**	**13.01**	**12.05**	**18.23**
	**Al**	**0.83**	**0.84**	**1.27**
	**Fe**	**0.66**	**0.59**	**0.97**
	**Zn**	**0.27**	**0.27**	**0.42**
	**P**	**31.59**	**30.45**	**47.63**
**Tree 2**	**Ca**	**88.01**	**86.06**	**132.70**
	**K**	**103.43**	**118.77**	**161.75**
	**Mg**	**53.33**	**51.85**	**78.75**
	**Mn**	**9.45**	**8.69**	**13.20**
	**Al**	**0.67**	**0.78**	**1.09**
	**Fe**	**0.56**	**0.54**	**0.84**
	**Zn**	**0.31**	**0.32**	**0.49**
	**P**	**34.39**	**33.81**	**52.81**
**Tree 3**	**Ca**	**79.88**	**79.44**	**110.80**
	**K**	**96.25**	**109.11**	**133.82**
	**Mg**	**45.18**	**44.81**	**62.34**
	**Mn**	**7.40**	**6.93**	**9.56**
	**Al**	**0.63**	**0.71**	**0.89**
	**Fe**	**0.54**	**0.54**	**0.74**
	**Zn**	**0.32**	**0.32**	**0.41**
	**P**	**29.43**	**29.29**	**41.11**
**Tree 4**	**Ca**	**111.10**	**111.81**	**153.78**
	**K**	**109.36**	**122.09**	**149.92**
	**Mg**	**48.02**	**49.45**	**67.21**
	**Mn**	**10.97**	**10.40**	**14.05**
	**Al**	**0.66**	**0.76**	**0.89**
	**Fe**	**0.51**	**0.47**	**0.65**
	**Zn**	**0.38**	**0.38**	**0.53**
	**P**	**33.00**	**32.36**	**45.18**

Supplemental Table 3. Raw data collected for the current year (CY) needles of red spruce total inorganic nutrients during all the collections are present here. With a few exceptions, these data are the mean of two analytical replicates. "na" denotes data not available.

Current Year (CY) Red Spruce				
**Collection Date**	**Elements ((µmol g-1 calc FW)**	**Dried-Ground-needles**	**Dried-needles**	**Wet-needles**
**6/24/2015**				
**Tree 1**	**Ca**	**11.78**	**12.17**	**11.59**
	**K**	**95.36**	**95.21**	**108.03**
	**Mg**	**11.31**	**11.92**	**13.03**
	**Mn**	**4.41**	**4.78**	**5.23**
	**Al**	**0.24**	**0.177**	**0.00**
	**Fe**	**0.12**	**0.092**	**0.10**
	**Zn**	**0.10**	**0.111**	**0.13**
	**P**	**25.36**	**26.45**	**29.72**
**9/29/2014**				
**Tree 1**	**Ca**	**41.68**	**na**	**40.41**
	**K**	**81.71**	**na**	**70.08**
	**Mg**	**26.99**	**na**	**23.38**
	**Mn**	**21.03**	**na**	**18.42**
	**Al**	**0.88**	**na**	**0.39**
	**Fe**	**0.22**	**na**	**0.16**
	**Zn**	**0.29**	**na**	**0.18**
	**P**	**33.64**	**na**	**30.49**
**Tree 2**	**Ca**	**17.31**	**na**	**15.54**
	**K**	**103.37**	**na**	**87.42**
	**Mg**	**20.38**	**na**	**17.56**
	**Mn**	**8.16**	**na**	**7.31**
	**Al**	**0.34**	**na**	**0.00**
	**Fe**	**0.24**	**na**	**0.15**
	**Zn**	**0.11**	**na**	**0.09**
	**P**	**33.42**	**na**	**28.69**
**Tree 3**	**Ca**	**39.82**	**na**	**37.25**
	**K**	**100.00**	**na**	**82.60**
	**Mg**	**30.74**	**na**	**27.24**
	**Mn**	**15.42**	**na**	**14.19**
	**Al**	**0.30**	**na**	**0.00**
	**Fe**	**0.30**	**na**	**0.21**
	**Zn**	**0.26**	**na**	**0.21**
	**P**	**35.79**	**na**	**31.42**
				**n=1**
**Tree 4**	**Ca**	**24.93**	**na**	**20.74**
	**K**	**95.02**	**na**	**80.77**
	**Mg**	**18.69**	**na**	**15.83**
	**Mn**	**8.22**	**na**	**7.06**
	**Al**	**0.69**	**na**	**0.08**
	**Fe**	**0.24**	**na**	**0.16**
	**Zn**	**0.12**	**na**	**0.11**
	**P**	**35.41**	**na**	**29.99**

4/29/2015				
Tree 1	Ca	37.42	37.94	47.83
	K	79.75	76.05	95.67
	Mg	23.25	23.01	29.25
	Mn	16.05	15.94	21.72
	Al	1.12	0.95	1.16
	Fe	0.09	0.13	0.10
	Zn	0.20	0.18	0.24
	P	31.35	30.94	40.07
Tree 2	Ca	43.18	44.62	46.84
	K	117.84	114.96	119.15
	Mg	29.02	29.24	30.78
	Mn	16.28	16.19	18.54
	Al	0.65	0.73	0.72
	Fe	0.14	0.18	0.12
	Zn	0.20	0.18	0.20
	P	37.81	37.84	40.11
Tree 3	Ca	56.63	58.44	69.70
	K	68.44	66.16	78.50
	Mg	34.67	34.59	41.73
	Mn	19.85	19.64	25.35
	Al	0.43	0.38	0.48
	Fe	0.18	0.19	0.17
	Zn	0.28	0.26	0.32
	P	36.01	36.62	43.31
Tree 4	Ca	27.31	27.16	34.38
	K	68.82	66.48	87.09
	Mg	18.80	18.44	23.18
	Mn	8.36	8.04	10.85
	Al	0.63	0.63	0.82
	Fe	0.12	0.16	0.12
	Zn	0.12	0.11	0.16
	P	27.25	27.37	34.75
2/26/2015				
Tree 1	Ca	49.55	50.81	54.24
	K	89.09	93.07	94.80
	Mg	31.85	31.87	34.01
	Mn	21.55	21.76	23.72
	Al	1.14	1.20	1.04
	Fe	0.18	0.15	0.18
	Zn	0.25	0.24	0.26
	P	37.98	38.73	40.43
Tree 2	Ca	33.74	35.90	40.85
	K	102.79	105.96	126.47
	Mg	24.63	25.29	29.35
	Mn	12.76	13.80	15.52
	Al	0.31	0.37	0.48
	Fe	0.21	0.18	0.20
	Zn	0.14	0.15	0.19
	P	33.79	35.35	40.24

**Tree 3**	**Ca**	**58.25**	**60.18**	**70.35**
	**K**	**77.54**	**80.45**	**94.51**
	**Mg**	**41.54**	**42.00**	**49.80**
	**Mn**	**21.00**	**21.53**	**25.73**
	**Al**	**0.56**	**0.58**	**0.61**
	**Fe**	**0.27**	**0.23**	**0.25**
	**Zn**	**0.32**	**0.32**	**0.38**
	**P**	**39.08**	**39.76**	**47.34**
**Tree 4**	**Ca**	**27.69**	**28.15**	**31.43**
	**K**	**89.14**	**93.00**	**106.33**
	**Mg**	**18.12**	**18.74**	**21.15**
	**Mn**	**9.40**	**9.55**	**11.50**
	**Al**	**0.80**	**0.92**	**0.84**
	**Fe**	**0.20**	**0.18**	**0.21**
	**Zn**	**0.13**	**0.12**	**0.15**
	**P**	**32.10**	**32.67**	**37.31**

Supplemental Table 4. Raw data collected for the previous year (PY) needles of red spruce total inorganic nutrients during all the collections are present here. With a few exceptions, these data are the mean of two analytical replicates. "na" denotes data not available.

Red Spruce Past Year (PY) Needles				
**Collection Date**	**Element ((µmol g-1 calc FW)**	**Dried-Ground-needles**	**Dried-needles**	**Wet-needles**
**6/24/2015**				
**Tree 1**	**Ca**	**35.80**	**37.29**	**37.61**
	**K**	**62.30**	**63.22**	**67.10**
	**Mg**	**13.00**	**13.35**	**13.83**
	**Mn**	**14.78**	**15.53**	**16.43**
	**Al**	**0.70**	**0.73**	**0.49**
	**Fe**	**0.21**	**0.19**	**0.19**
	**Zn**	**0.19**	**0.19**	**0.20**
	**P**	**23.44**	**23.86**	**26.16**
**9/29/2014**				
**Tree 1**	**Ca**	**33.62**	**32.49**	**32.98**
	**K**	**68.45**	**67.78**	**66.94**
	**Mg**	**12.59**	**12.67**	**12.24**
	**Mn**	**13.34**	**13.38**	**13.36**
	**Al**	**0.65**	**0.57**	**0.25**
	**Fe**	**0.19**	**0.12**	**0.15**
	**Zn**	**0.18**	**0.17**	**0.17**
	**P**	**25.30**	**25.24**	**25.24**
**Tree 2**	**Ca**	**18.68**	**na**	**19.85**
	**K**	**63.16**	**na**	**64.82**
	**Mg**	**10.94**	**na**	**10.92**
	**Mn**	**6.95**	**na**	**6.94**
	**Al**	**0.86**	**na**	**0.42**
	**Fe**	**0.28**	**na**	**0.16**
	**Zn**	**0.08**	**na**	**0.07**
	**P**	**18.59**	**na**	**19.66**
**Tree 3**	**Ca**	**55.67**	**na**	**57.82**
	**K**	**78.57**	**na**	**81.53**
	**Mg**	**23.01**	**na**	**23.30**
	**Mn**	**17.30**	**na**	**17.81**
	**Al**	**0.60**	**na**	**0.26**
	**Fe**	**0.27**	**na**	**0.20**
	**Zn**	**0.31**	**na**	**0.28**
	**P**	**31.12**	**na**	**31.21**
**Tree 4**	**Ca**	**33.50**	**na**	**33.64**
	**K**	**65.98**	**na**	**63.48**
	**Mg**	**13.69**	**na**	**13.22**
	**Mn**	**9.22**	**na**	**8.91**
	**Al**	**1.49**	**na**	**1.03**

	Fe	0.28	na	0.18
	Zn	0.34	na	0.11
	P	22.29	na	22.40
4/29/2015				
Tree 1	Ca	41.02	42.73	45.07
	K	85.29	83.44	89.25
	Mg	16.64	16.89	17.95
	Mn	14.41	14.68	16.96
	Al	0.87	0.92	0.82
	Fe	0.15	0.16	0.10
	Zn	0.24	0.22	0.24
	P	26.81	27.43	29.15
Tree 2	Ca	63.93	65.09	70.83
	K	99.36	96.35	105.96
	Mg	24.64	24.55	26.96
	Mn	18.85	19.32	22.84
	Al	0.93	0.99	1.10
	Fe	0.19	0.21	0.17
	Zn	0.24	0.21	0.24
	P	27.04	26.91	29.77
Tree 3	Ca	58.55	61.11	66.67
	K	75.72	74.60	78.50
	Mg	26.07	26.12	27.88
	Mn	17.10	17.64	20.88
	Al	0.42	0.41	0.29
	Fe	0.23	0.26	0.19
	Zn	0.24	0.25	0.27
	P	29.52	30.45	31.31
Tree 4	Ca	39.66	39.60	45.37
	K	67.28	66.04	73.63
	Mg	15.23	15.22	17.08
	Mn	9.52	9.46	11.44
	Al	1.01	1.07	1.04
	Fe	0.21	0.17	0.16
	Zn	0.14	0.13	0.15
	P	22.09	22.07	24.64
2/26/2015				
Tree 1	Ca	37.38	36.72	43.12
	K	91.32	92.68	107.50
	Mg	12.40	12.17	14.47
	Mn	13.46	14.19	16.58
	Al	0.85	0.87	1.02
	Fe	0.21	0.19	0.22
	Zn	0.20	0.20	0.27
	P	32.00	32.72	38.22
Tree 2	Ca	38.64	40.35	46.20
	K	78.12	81.35	94.60
	Mg	16.40	16.94	19.95
	Mn	12.32	12.99	15.07
	Al	0.67	0.69	0.82

	**Fe**	**0.22**	**0.21**	**0.25**
	**Zn**	**0.13**	**0.12**	**0.14**
	**P**	**22.49**	**23.69**	**27.14**
**Tree 3**	**Ca**	**74.45**	**75.85**	**78.50**
	**K**	**112.27**	**115.51**	**122.86**
	**Mg**	**30.60**	**31.39**	**32.45**
	**Mn**	**21.26**	**21.54**	**22.12**
	**Al**	**1.59**	**1.51**	**1.69**
	**Fe**	**0.32**	**0.33**	**0.33**
	**Zn**	**0.35**	**0.36**	**0.38**
	**P**	**41.60**	**42.06**	**44.48**
**Tree 4**	**Ca**	**40.24**	**41.25**	**49.26**
	**K**	**78.51**	**80.40**	**97.38**
	**Mg**	**17.37**	**17.36**	**21.10**
	**Mn**	**13.16**	**13.67**	**16.61**
	**Al**	**1.39**	**1.51**	**1.77**
	**Fe**	**0.23**	**0.23**	**0.26**
	**Zn**	**0.14**	**0.14**	**0.16**
	**P**	**26.25**	**26.77**	**31.89**

Supplemental Table 5. Raw data collected for the current year (CY) needles of white pine total inorganic nutrients during all the collections are present here. With a few exceptions, these data are the mean of two analytical replicates. "na" denotes data not available.

Current Year (CY) White Pine				
**Collection Date**	**Element (µmol g^{-1} calc FW)**	**Dried-Ground neeles**	**Dried-needles**	**Wet-needles**
**6/24/2014**				
**Tree 1**	**Ca**	**9.82**	**9.88**	**9.55**
	**K**	**90.52**	**89.32**	**99.04**
	**Mg**	**11.13**	**11.04**	**12.16**
	**Mn**	**0.655**	**0.640**	**0.580**
	**Al**	**0.420**	**0.369**	**0.004**
	**Fe**	**0.224**	**0.171**	**0.189**
	**Zn**	**0.195**	**0.195**	**0.207**
	**P**	**28.37**	**28.57**	**30.92**
**9/29/2014**				
**Tree 1**	**Ca**	**44.23**	**na**	**38.48**
	**K**	**44.16**	**na**	**37.62**
	**Mg**	**33.20**	**na**	**28.72**
	**Mn**	**2.96**	**na**	**2.31**
	**Al**	**3.68**	**na**	**2.65**
	**Fe**	**0.38**	**na**	**0.24**
	**Zn**	**0.78**	**na**	**0.46**
	**P**	**23.28**	**na**	**19.98**
**Tree 2**	**Ca**	**43.95**	**na**	**41.29**
	**K**	**50.82**	**na**	**54.33**
	**Mg**	**28.63**	**na**	**28.41**
	**Mn**	**3.79**	**na**	**2.30**
	**Al**	**3.68**	**na**	**1.98**
	**Fe**	**0.57**	**na**	**0.36**
	**Zn**	**0.31**	**na**	**0.40**
	**P**	**23.94**	**na**	**26.01**
**Tree 3**	**Ca**	**43.95**	**44.28**	**38.73**
	**K**	**50.82**	**48.92**	**42.78**
	**Mg**	**28.63**	**28.74**	**23.87**
	**Mn**	**3.79**	**3.76**	**3.15**
	**Al**	**3.68**	**3.51**	**2.49**
	**Fe**	**0.57**	**0.40**	**0.34**
	**Zn**	**0.31**	**0.28**	**0.26**
	**P**	**23.94**	**24.70**	**20.77**
**Tree 4**	**Ca**	**24.51**	**na**	**18.50**
	**K**	**82.20**	**na**	**66.08**
	**Mg**	**31.42**	**na**	**25.27**
	**Mn**	**2.28**	**na**	**1.83**
	**Al**	**3.10**	**na**	**1.88**
	**Fe**	**0.58**	**na**	**0.32**
	**Zn**	**0.71**	**na**	**0.33**

	P	33.51	na	26.14
02/26/15				
Tree 1	Ca	46.23	45.82	51.92
	K	47.88	48.74	55.10
	Mg	29.72	29.99	34.21
	Mn	2.58	2.72	3.09
	Al	2.69	2.62	2.92
	Fe	0.31	0.26	0.30
	Zn	0.61	0.59	0.67
	P	25.01	25.05	28.91
Tree 2	Ca	58.51	58.34	70.58
	K	57.57	59.21	71.56
	Mg	32.91	32.98	41.39
	Mn	3.31	3.20	3.97
	Al	3.16	3.14	3.72
	Fe	0.41	0.37	0.44
	Zn	0.62	0.60	0.74
	P	29.56	29.74	36.49
Tree 3	Ca	46.50	46.75	54.54
	K	71.41	72.30	84.96
	Mg	33.03	33.15	39.13
	Mn	2.26	2.40	2.81
	Al	3.85	3.93	4.73
	Fe	0.37	0.36	0.43
	Zn	0.63	0.62	0.75
	P	28.52	28.90	34.14
Tree 4	Ca	24.06	24.51	24.66
	K	78.03	80.61	83.05
	Mg	24.31	24.94	25.78
	Mn	2.10	2.15	2.18
	Al	3.03	3.20	3.06
	Fe	0.36	0.34	0.36
	Zn	0.32	0.30	0.32
	P	31.26	31.56	33.30
04/29/15				
Tree 1	Ca	47.08	47.62	50.28
	K	53.61	52.86	55.19
	Mg	34.69	34.93	37.26
	Mn	2.32	2.40	2.84
	Al	3.82	3.79	3.63
	Fe	0.42	0.40	0.36
	Zn	0.76	0.72	0.79
	P	26.25	25.95	27.88
Tree 2	Ca	51.98	52.37	57.80
	K	70.62	68.23	76.33
	Mg	30.38	30.09	33.22
	Mn	2.37	2.35	2.77
	Al	2.55	2.55	2.59

	Fe	0.33	0.31	0.29
	Zn	0.51	0.49	0.56
	P	29.50	29.70	33.23
Tree 3	Ca	42.29	42.42	46.07
	K	68.84	67.22	72.61
	Mg	27.62	27.67	30.06
	Mn	3.65	3.51	4.13
	Al	4.85	4.79	5.03
	Fe	0.35	0.31	0.30
	Zn	0.46	0.44	0.50
	P	27.79	27.77	30.13
Tree 4	Ca	34.61	35.45	37.42
	K	75.04	73.07	79.12
	Mg	26.19	26.45	27.93
	Mn	2.64	2.77	3.06
	Al	3.37	3.83	3.41
	Fe	0.39	0.35	0.34
	Zn	0.46	0.45	0.51
	P	28.80	28.74	32.00

Supplemental Table 6. Raw data collected for the previous year (PY) needles of white pine total inorganic nutrients during all the collections are present here. These data are the mean of two analytical replicates. "na" denotes data not available. **These needles were shed off at the end of winter because of needle-cast fungal infection and thus prest of the collections could not be made through winter and the following year.

White Pine Past Year (PY) Needles Digests				
**Collection Date**	**Element ((µmol g-1 calc FW)**	**Dried-Ground neeles**	**Dried-needles**	**Wet-needles**
**6/24/2015**		**Mean**	**Mean**	**Mean**
**Tree 1**	**Ca**	**42.55**	**42.09**	**46.19**
	**K**	**61.86**	**64.46**	**68.68**
	**Mg**	**23.35**	**23.77**	**25.93**
	**Mn**	**1.71**	**1.68**	**1.95**
	**Al**	**3.01**	**3.04**	**3.38**
	**Fe**	**0.34**	**0.30**	**0.41**
	**Zn**	**0.43**	**0.45**	**0.48**
	**P**	**24.12**	**24.21**	**26.56**
