# Supplementary material for: Is foliar tissue drying and grinding required for reliable and reproducible extraction of total inorganic nutrients? A comparative study of three tissue preparation methods
Source: Front Plant Sci. 2022 Nov 18;13:1012764. doi: 10.3389/fpls.2022.1012764 (PMC9716281; doi:10.3389/fpls.2022.1012764)
Supplement: Supplementary file 2 [file DataSheet_2.pdf]

Supplemental Materials

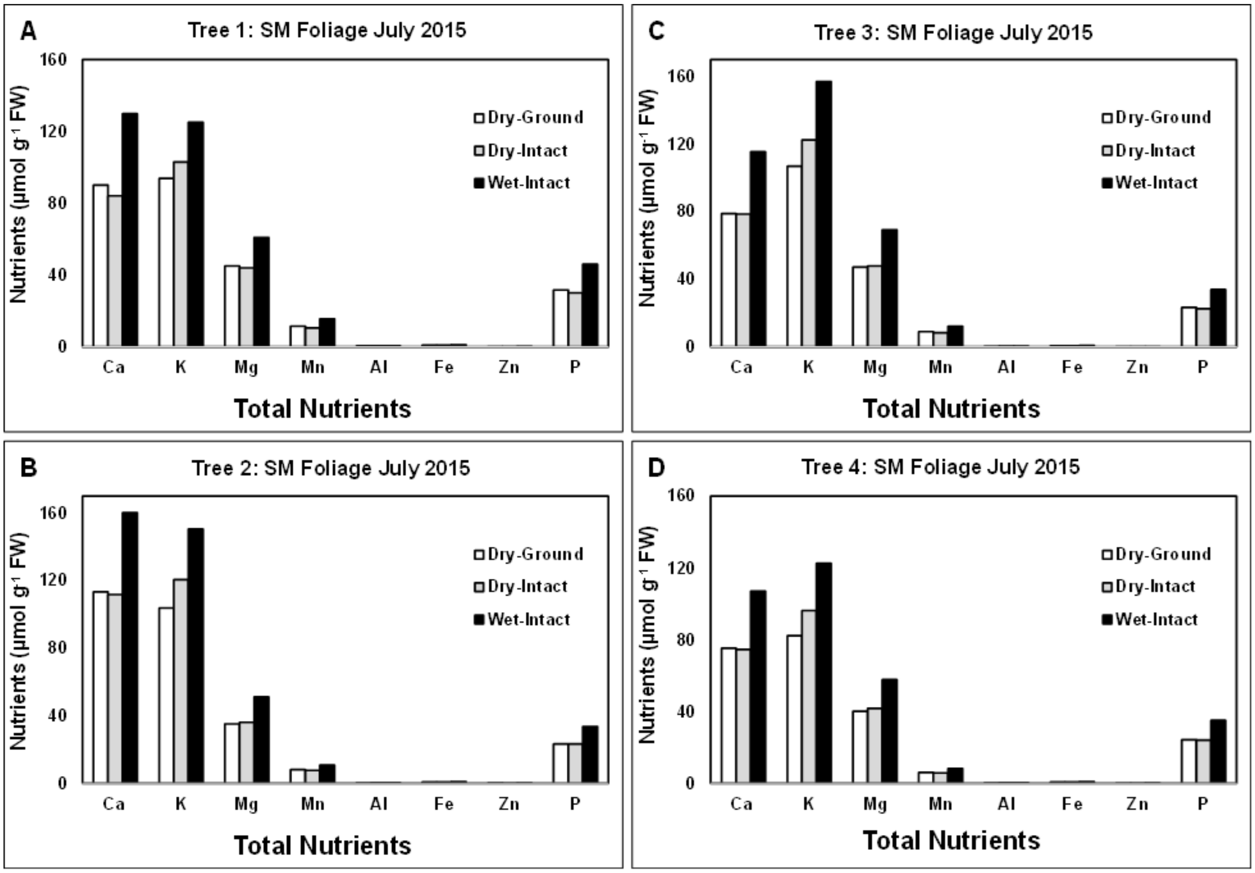

Supplemental Figure 1. Comparison of three tissue preparation methods for the extraction of total inorganic nutrients from the foliage of four replicate trees of sugar maple collected in July 2015: Tree 1 (A); Tree 2 (B); Tree 3 (C); and Tree 4 (D).

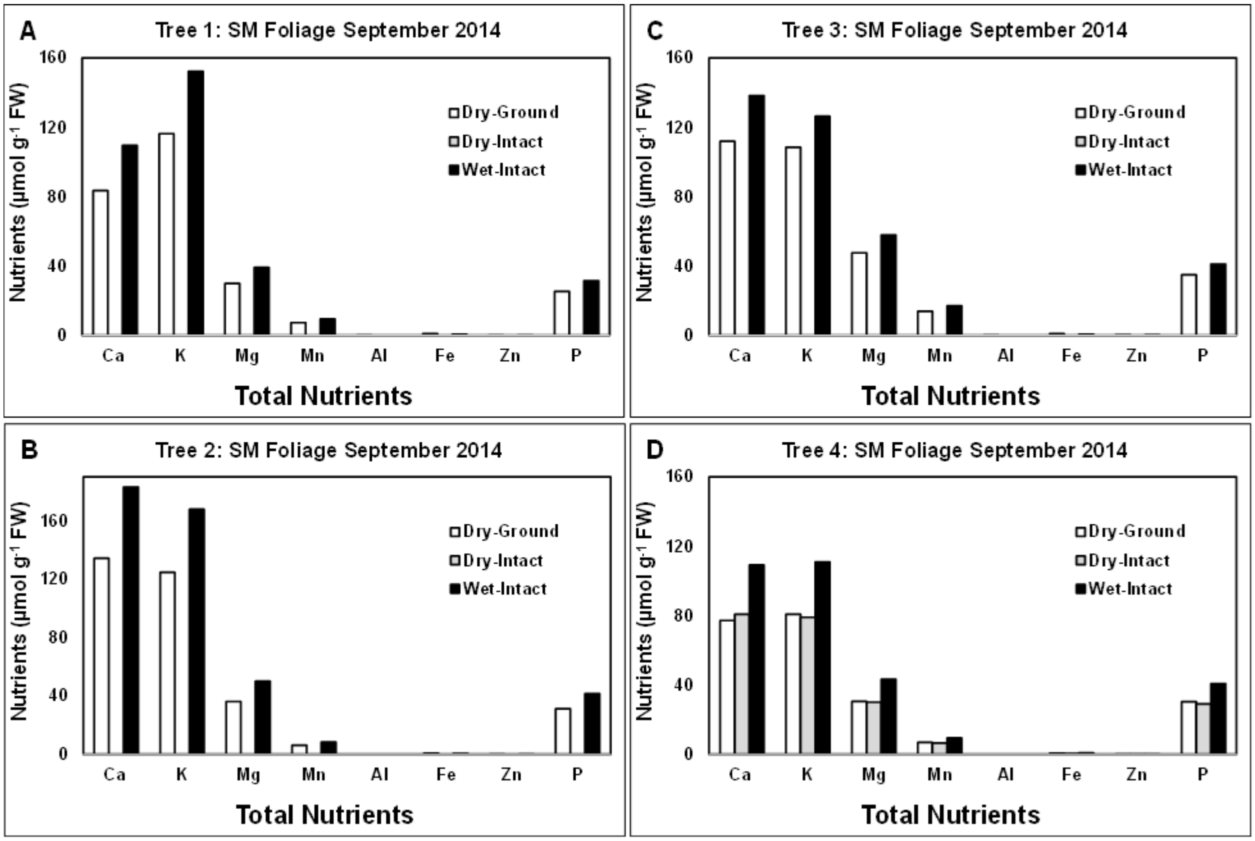

Supplemental Figure 2. Comparison of three tissue preparation methods for extraction of total inorganic nutrients from the foliage of four replicate trees of sugar maple collected in September 2014: Tree 1 (A); Tree 2 (B); Tree 3 (C); and Tree 4 (D). *Dry-Intact data are unavailable for trees 1, 2, and 3.

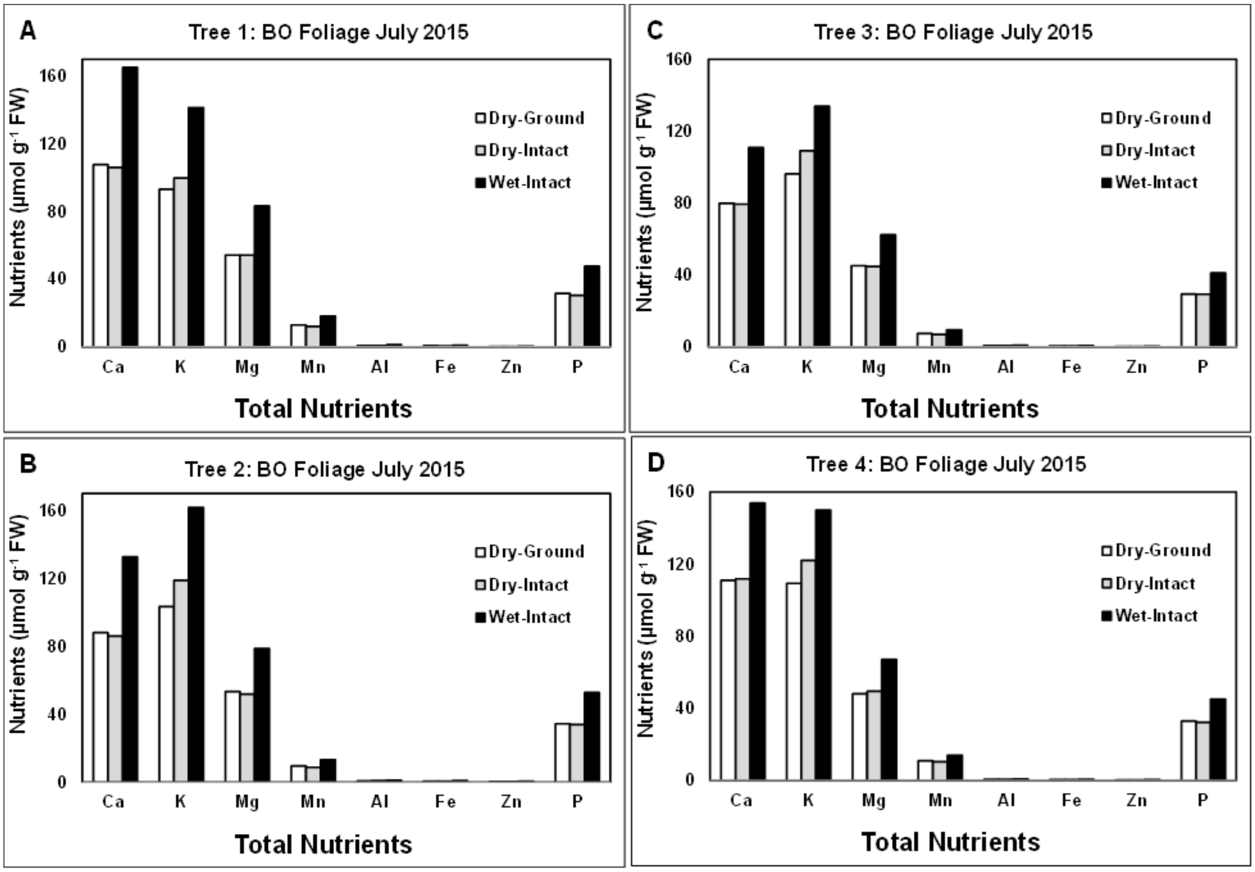

Supplemental Figure 3. Comparison of three tissue preparation methods for the extraction of total inorganic nutrients from the foliage of four replicate trees of black oak collected in July 2015: Tree 1 (A); Tree 2 (B); Tree 3 (C); and Tree 4 (D).

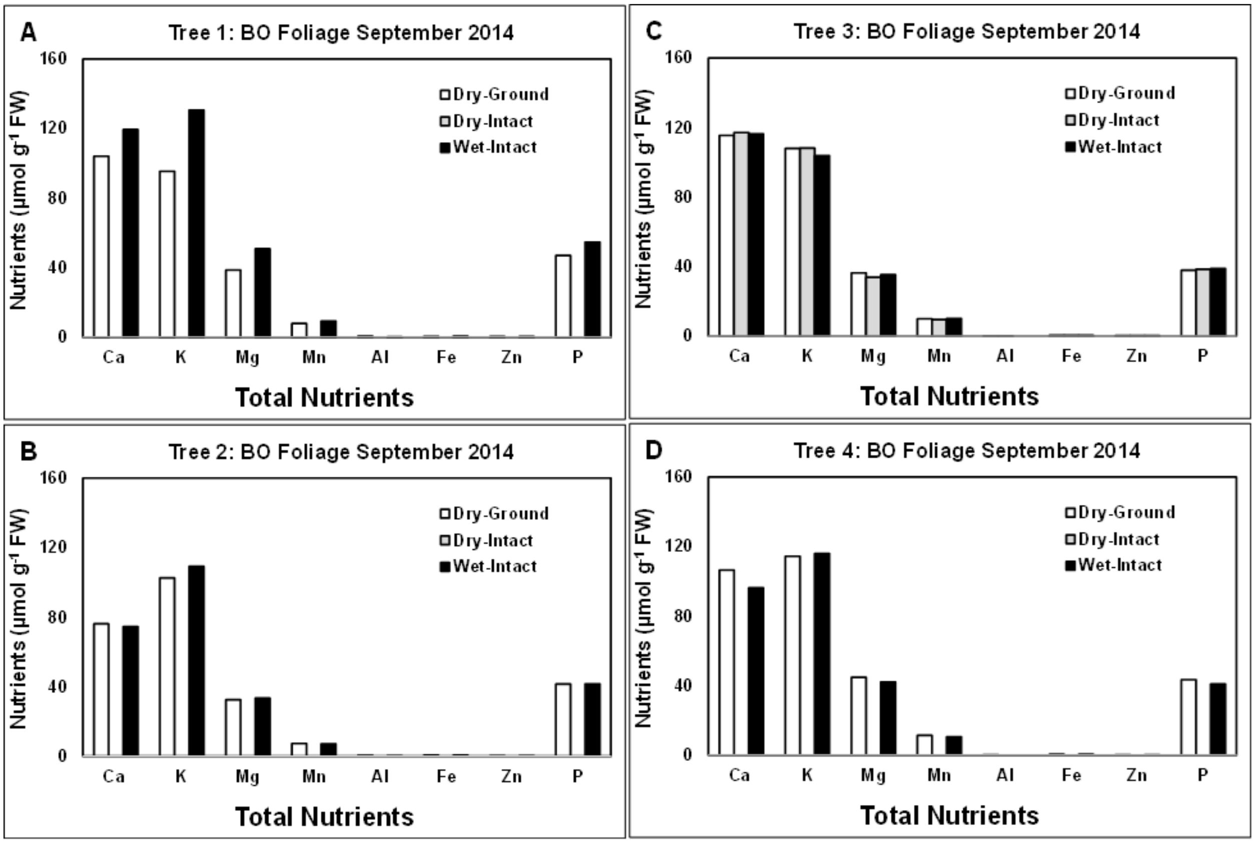

Supplemental Figure 4. Comparison of three tissue preparation methods for extraction of total inorganic nutrients from the foliage of four replicate trees of black oak collected in September 2014: Tree 1 (A); Tree 2 (B); Tree 3 (C); and Tree 4 (D). *Dry-Intact data are unavailable for trees 1, 2, and 3.

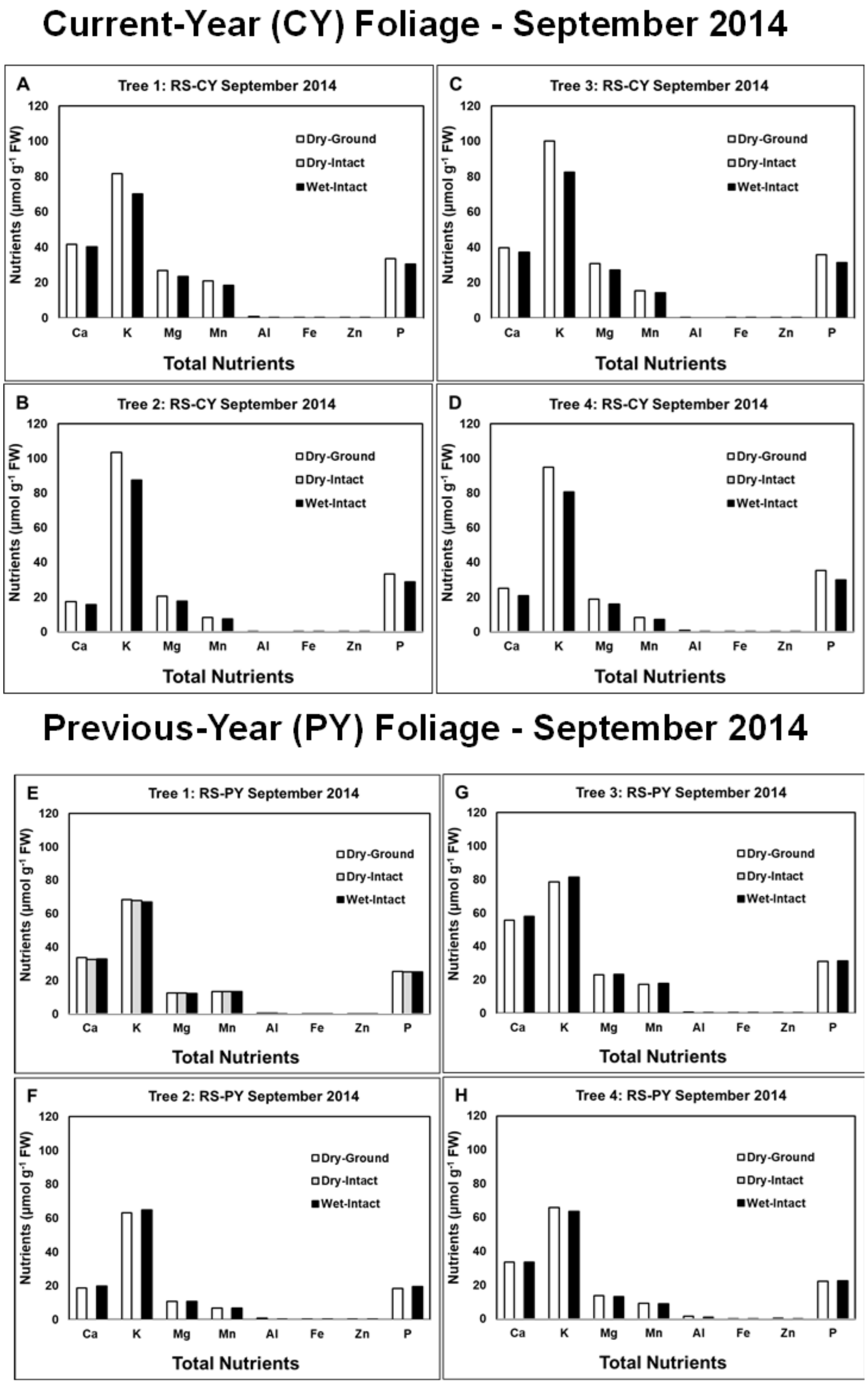

Supplemental Figure 5. Comparison of three tissue preparation methods for extraction of total inorganic nutrients from four replicate trees of red spruce collected in September 2014: CY foliage (A-D) and PY foliage (E-H) collected from the same branchlet. Data are: Tree 1 (A, E); Tree 2 (B, F); Tree 3 (C, G); and Tree 4 (D, H). *Dry-Intact data are unavailable for trees 2, 3, and 4.

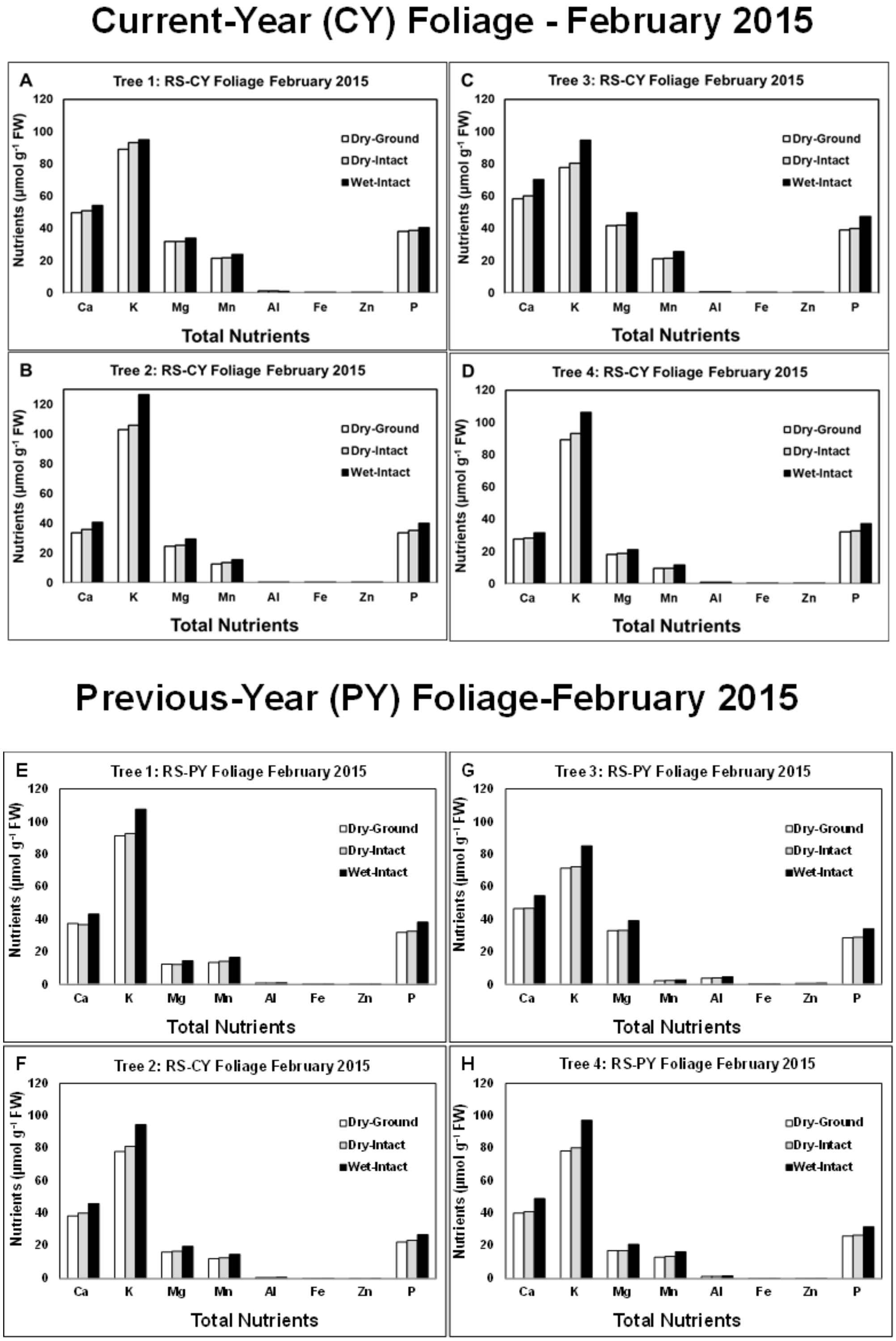

Supplemental Figure 6 Comparison of three tissue preparation methods for extraction of total inorganic nutrients from four replicate trees of red spruce collected in February 2015: CY foliage (A-D) and PY foliage (E-H) were collected from the same branchlet. Data are: Tree 1 (A, E); Tree 2 (B, F); Tree 3 (C, G); and Tree 4 (D, H).

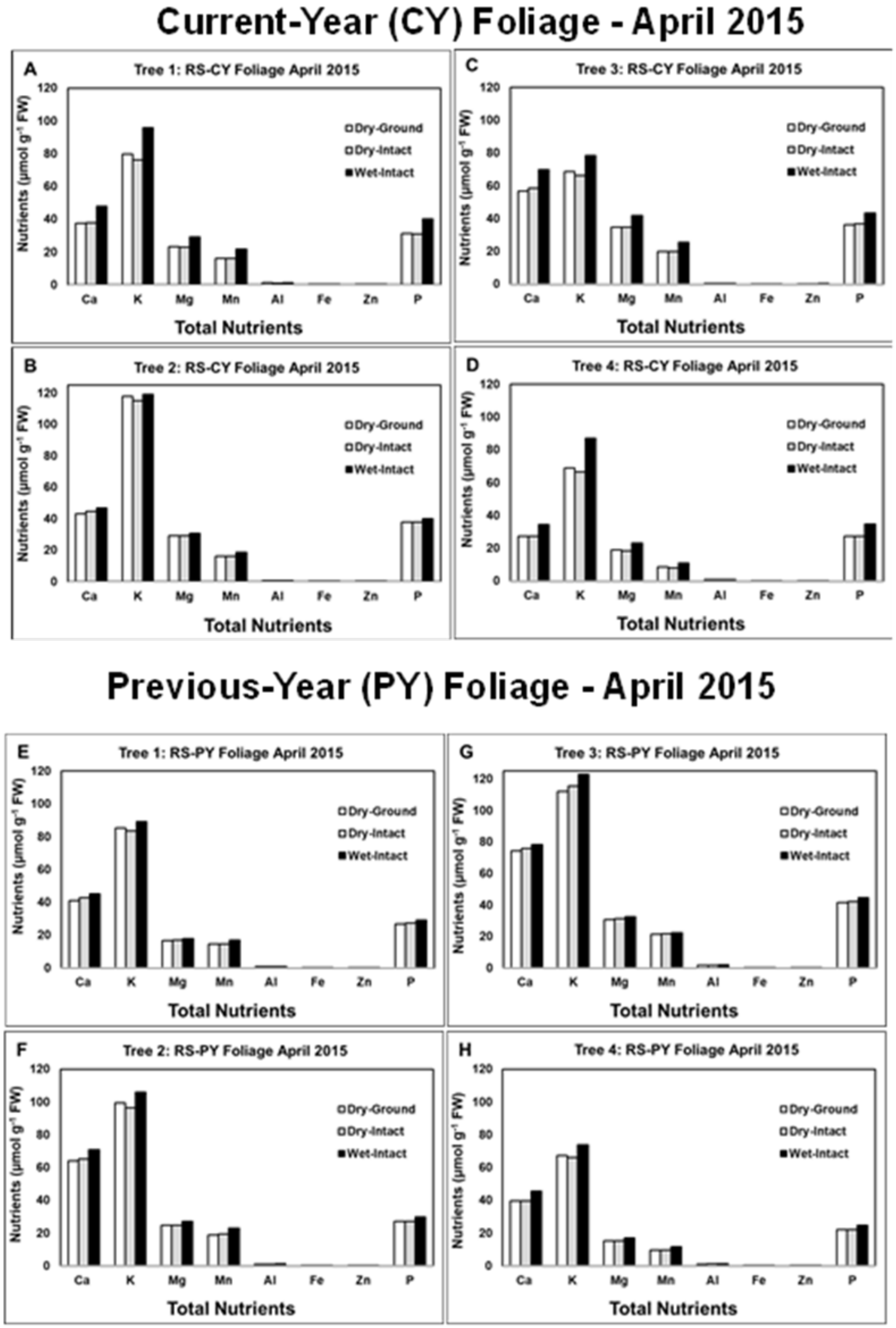

Supplemental Figure 7. Comparison of three tissue preparation methods for the extraction of total inorganic nutrients from the foliage of four replicate trees of red spruce CY (A-D) and PY (E-H) foliage collected from the same branchlet in April 2015: Tree 1 (A, E); Tree 2 (B, F); Tree 3 (C, G); and Tree 4 (D, H).

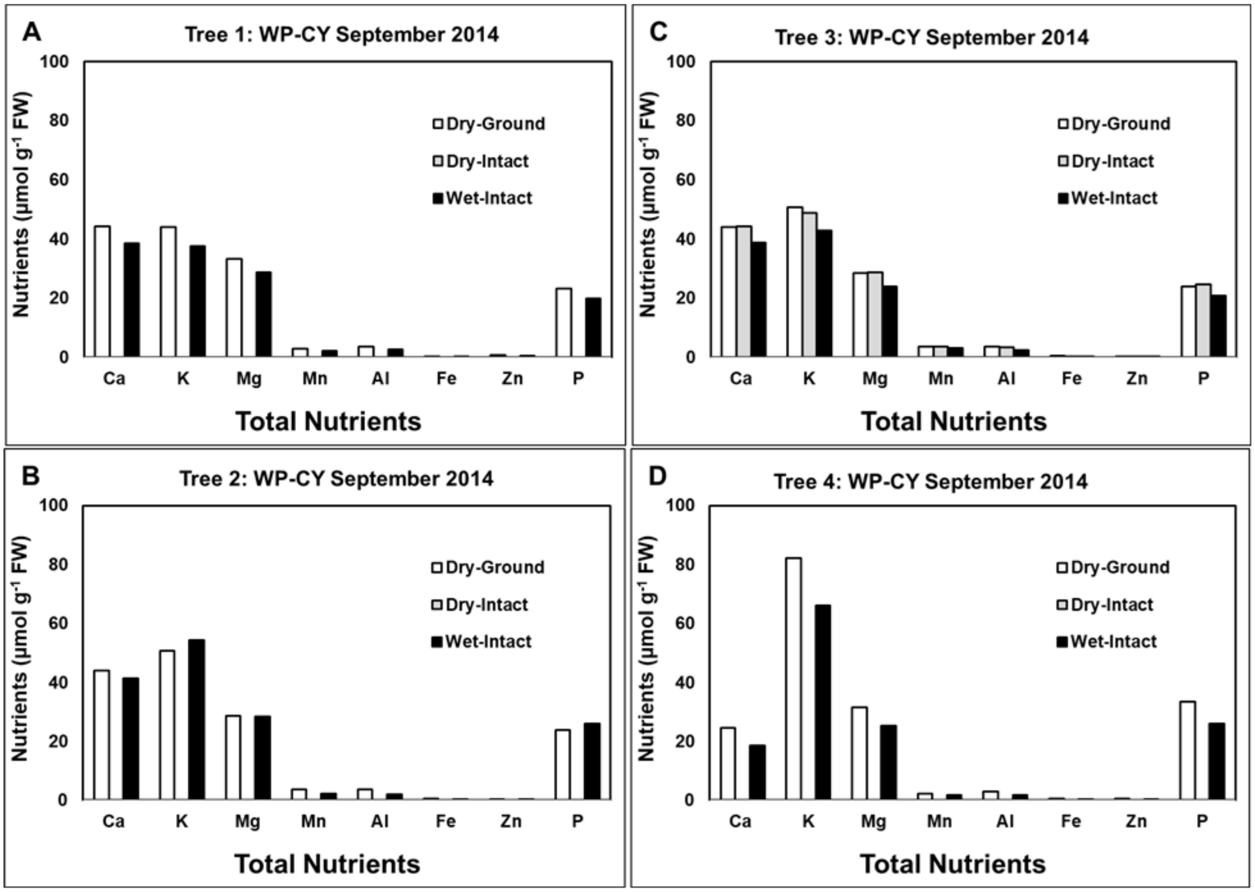

Supplemental Figure 8. Comparison of three different tissue preparation methods for extraction of total inorganic nutrients from CY foliage of four replicate trees of white pine trees collected in September 2014. Data are: Tree 1 (A); Tree 2 (B); Tree 3 (C); and Tree 4 (D). *Dry-Intact data are unavailable for trees 1, 2, and 4.

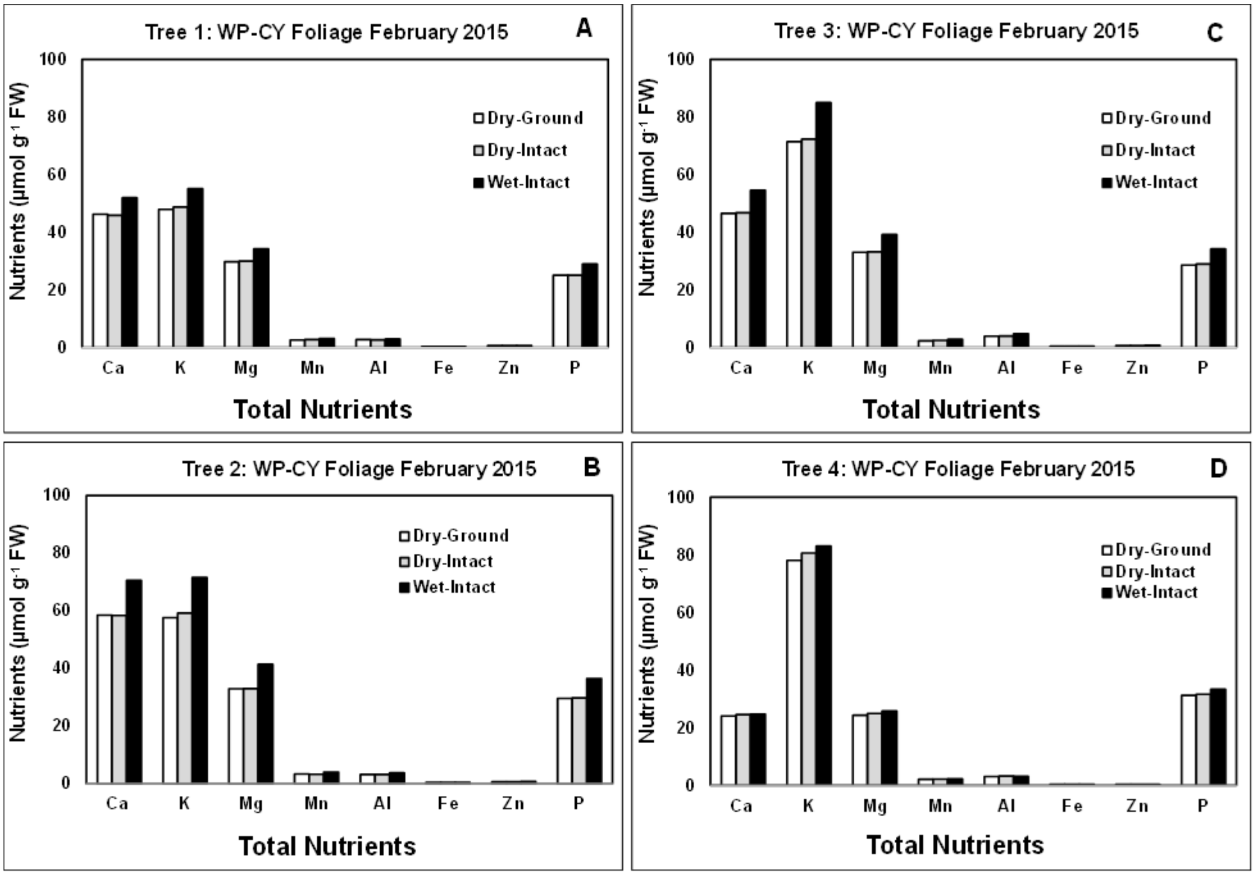

Supplemental Figure 9. Comparison of three different tissue preparation methods for extraction of total inorganic nutrients from CY foliage of four replicate trees of white pine trees collected in February 2015: Tree 1 (A); Tree 2 (B); Tree 3 (C); and Tree 4 (D).

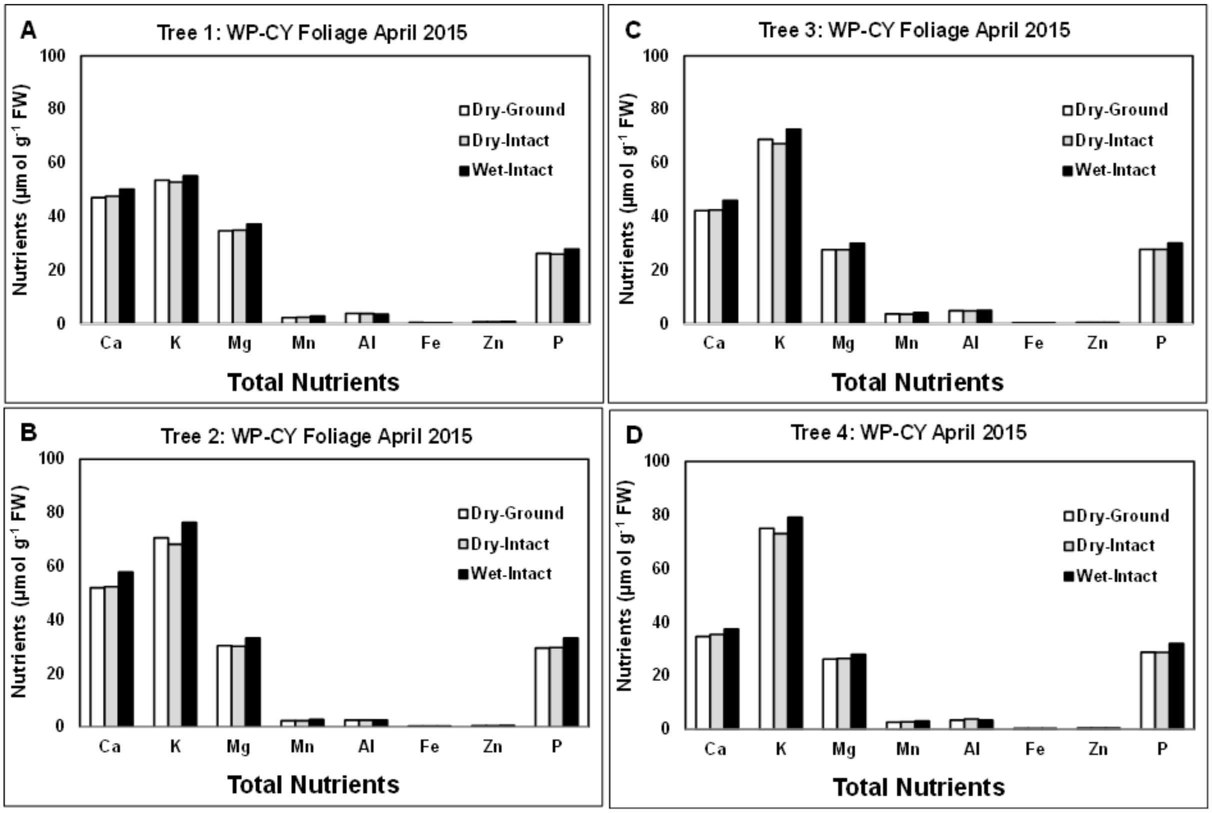

Supplemental Figure 10. Comparison of three tissue preparation methods for extraction of total inorganic nutrients from CY foliage collected in April 2015 from four replicate trees of white pine: Tree 1 (A); Tree 2 (B); Tree 3 (C); and Tree 4 (D).
